# Performance of the UK Prospective Diabetes Study Outcomes Model 2 in a Contemporary UK Type 2 Diabetes Trial Cohort

**DOI:** 10.1016/j.jval.2021.09.005

**Published:** 2022-03

**Authors:** Mi Jun Keng, Jose Leal, Marion Mafham, Louise Bowman, Jane Armitage, Borislava Mihaylova

**Affiliations:** 1 Health Economics Research Centre, Nuffield Department of Population Health, University of Oxford, Oxford, England, UK; 2 British Heart Foundation Centre of Research Excellence, Oxford, England, UK; 3 Clinical Trial Service Unit and Epidemiological Studies Unit, Nuffield Department of Population Health, University of Oxford, Oxford, England, UK; 4 Medical Research Council Population Health Research Unit, Nuffield Department of Population Health, University of Oxford, Oxford, England, UK; 5 Wolfson Institute of Population Health, Queen Mary University of London, London, England, UK

**Keywords:** ASCEND, type 2 diabetes, UK Prospective Diabetes Study outcomes model, validation

## Abstract

**Objectives:**

The UK Prospective Diabetes Study (UKPDS) Outcomes Model (UKPDS-OM) developed using 30-year (1977-2007) data from the UKPDS is widely used for health outcomes’ projections and economic evaluations of therapies for patients with type 2 diabetes (T2D). Nevertheless, its reliability for contemporary UK T2D populations is unclear. We assessed the performance of version 2 of the model (UKPDS-OM2) using data from A Study of Cardiovascular Events in Diabetes (ASCEND), which followed participants with diabetes in the UK between 2005 and 2017.

**Methods:**

The UKPDS-OM2 was used to predict the occurrence of myocardial infarction (MI), other ischemic heart disease, stroke, cardiovascular (CV) death, and other death among the 14 569 participants with T2D in ASCEND, all without previous CV disease at study entry. Calibration (comparison of predicted and observed year-on-year cumulative incidence over 10 years) and discrimination (c-statistics) of the model were assessed for each endpoint. The percentage error in event rates at year 7 (mean duration of follow up) was used to quantify model bias.

**Results:**

The UKPDS-OM2 substantially overpredicted MI, stroke, CV death, and other death over the 10-year follow-up period (by 149%, 42%, 269%, and 52%, respectively, at year 7). Discrimination of the model for MI and other ischemic heart disease (c-statistics 0.58 and 0.60, respectively) was poorer than that for other outcomes (c-statistics ranging from 0.66 to 0.72).

**Conclusions:**

The UKPDS-OM2 substantially overpredicted risks of key CV outcomes and death in people with T2D in ASCEND. Appropriate adjustments or a new model may be required for assessments of long-term effects of treatments in contemporary T2D cohorts.

## Highlights

• There is uncertainty over the accuracy of predictions by the UK Prospective Diabetes Study Outcomes Model 2 (UKPDS-OM2), developed using patient data collected from 1977 to 2007, in contemporary diabetes cohort.
• This study demonstrates that the UKPDS-OM2 overpredicts the risks of myocardial infarction, stroke, heart failure, and deaths over 10 years of follow up in A Study of Cardiovascular Events in Diabetes (ASCEND), one of the largest trials in people with diabetes in the United Kingdom that followed participants from 2005 to 2017. In particular, the performance of the UKPDS-OM2 was found to be poorer in older patients who received a diagnosis of diabetes at an older age.
• Calibration of risk equations in the UKPDS-OM2 or estimation of new risk equations is needed to predict long-term outcomes for clinical or economic analyses in contemporary cohorts such as in ASCEND.

## Introduction

Decision-analytic modeling is commonly used in healthcare to synthesize clinical and economic data to evaluate health outcomes and costs of healthcare interventions.^1^ These models are used as decision-support tools to inform decisions on reimbursement of new drugs, allocation of resources between competing interventions, estimation of the budget impact of new interventions, and other health policy questions. Such models can also be used to extrapolate short-term clinical trial data to evaluate the long-term effects of interventions on health outcomes and healthcare costs and to inform cost-effectiveness analyses.^2^^,^^3^ This is especially relevant for type 2 diabetes (T2D), a chronic disease associated with a range of macrovascular and microvascular complications that develop over a lifetime and are major drivers of long-term survival, deterioration in quality of life, and healthcare costs.^4^, ^5^, ^6^ Consequently, such decision-analytic models need to provide accurate outcome estimates and, therefore, warrant validation, as recommended by published guidelines.^2^^,^^7^ External validation of a model is particularly important to ensure its suitability for patient cohorts other than the one used to develop the model.^8^^,^^9^

The UK Prospective Diabetes Study (UKPDS) Outcomes Model (UKPDS-OM) is a stochastic patient-level simulation model that uses the risk factor profiles of patients with T2D to predict the occurrence of diabetes-related macrovascular and microvascular complications over patients’ lifetimes and at the same time to quantify their (quality-adjusted) life-years and healthcare costs.^10^^,^^11^ It is the National Institute for Health and Care Excellence’s model of choice for health technology assessments of diabetes treatments,^3^ and its risk equations are also used in several other commonly used diabetes simulation model.^12^, ^13^, ^14^, ^15^ Version 1 of the model was developed using data collected in the UKPDS, which randomized participants between 1977 and 1991, and followed them until 1997 (median follow-up 10.3 years).^10^ Temporal validation of the model using data collected during the 10-year post-trial monitoring period found that the model overpredicted the 10-year risks of heart failure and mortality and underpredicted the 10-year risks of stroke and amputation.^16^ Risk equations in the original model were subsequently updated including also the post-trial follow-up data (overall median follow-up 17.6 years) and version 2 of the model (UKPDS-OM2) was released.^11^ Internal validation of the UKPDS-OM2 demonstrated good agreement between simulated and observed outcomes among UKPDS patients over a period of 25 years. External validations of the UKPDS-OM2 have been performed in several more recent North American cohorts,^17^^,^^18^ a German cohort,^19^ an Italian cohort and a Dutch cohort.^20^ All studies report overprediction of mortality. This is consistent with observations in the report from the latest Mount Hood Diabetes Challenge, a forum for diabetes modelers to compare model performance, which found the UKPDS-OM2 and other diabetes model based on risk equations of the UKPDS-OM2 to overestimate cardiovascular (CV) mortality, although the results were generated using simulated patient cohorts instead of individual patient-level data.^21^ Overprediction of myocardial infarction (MI) was also observed in the North American, Italian, and Dutch cohorts, but not in the German cohort. The performance of the model can vary among countries because of differences in healthcare systems and health of the population in general (reflected by differences in life expectancy, mortality rates, and rates of T2D-related complications).^22^ In a guideline for good research practice, the issue of transferability of models across different settings was recognized, and it was suggested that careful consideration needs to be taken to ensure relevance of models in the population of interest.^23^ Findings from a previous study using The Health Improvement Network database in the United Kingdom suggest that the UKPDS-OM2 overpredicts MI, but no direct comparison between predicted and observed risk was presented and no results for mortality were reported.^24^ It remains unclear whether the UKPDS-OM2 can predict well in a contemporary UK cohort. Hence, we took the opportunity of the large long-term randomized trial A Study of Cardiovascular Events in Diabetes (ASCEND),^25^ which included 15 480 participants with diabetes recruited in the United Kingdom followed for an average of 7.4 years to assess the performance of the UKPDS-OM2.

## Methods

### Validation Cohort

Details of the ASCEND trial design and the main results have been previously reported.^25^, ^26^, ^27^ Briefly, ASCEND was a 2 × 2 factorial design trial that randomized 15 480 participants with established diabetes mellitus (both type 1 and type 2) but without diagnosed CV disease (CVD) to 100 mg aspirin daily or matching placebo and, separately, to 1 g capsule containing omega-3 fatty acids daily or placebo. Participants were recruited between 2005 and 2011 and followed for an average of 7.4 years until 2017. Among ASCEND participants, only those with T2D formed the validation cohort because UKPDS-OM2 was developed for predicting outcomes in patients with T2D.

### UKPDS Outcomes Model Version 2

The UKPDS-OM2 is a patient-level simulation that takes patient demographics (eg, age, sex, ethnicity), diabetes duration, history of T2D-related complications, and risk factor progression over time (all inputs required listed in Appendix Table 1 in Supplemental Materials found at https://doi.org/10.1016/j.jval.2021.09.005) to predict the occurrence of macrovascular events (MI, other ischemic heart disease [IHD], heart failure, and stroke), microvascular events (lower limb amputation, blindness, end-stage renal failure, and lower extremity ulcer), and deaths (all-cause death and CV death [defined as death from MI, other IHD, heart failure, or stroke]) (definition of each endpoint in Appendix Table 2 in Supplemental Materials found at https://doi.org/10.1016/j.jval.2021.09.005). The UKPDS-OM2 is an improved update from the UKPDS-OM1,^10^ with risk equations re-estimated using an additional 38 000 patient-years of observational data and including several additional outcomes.^11^

### Missing Data

Low-density lipoprotein cholesterol, hemoglobin, and white blood cell count measurements were not available in ASCEND. External data available to the authors were used to establish the relationships among these characteristics and other patient characteristics at baseline, which were used to impute these missing values.^28^^,^^29^ Other missing baseline data were replaced with the average value (for continuous variables) or most frequent occurrence (for binary variables) across 60 data sets created using the multivariate imputation by the chained equation method. Further details about the imputation of missing baseline data can be found in Appendix Section 1 in Supplemental Materials found at https://doi.org/10.1016/j.jval.2021.09.005.

Additionally, the UKPDS-OM2 requires the specification of risk factor progression over time. Nevertheless, biomarkers were collected only at baseline for most participants in ASCEND. Hence, the risk factor progression equations in the UKPDS-OM2^30^ were used to project risk factors in subsequent years.

### Endpoints

The key endpoints included were deaths (CV death as defined in UKPDS-OM2 and other death), MI, other IHD (includes angina, death from coronary heart disease not because of MI and coronary revascularization) and stroke, because these endpoints were rigorously sought, recorded, and adjudicated in ASCEND. Heart failure and amputation were patient reported and not adjudicated, so these were presented for exploratory purposes. Blindness, end-stage renal failure, and lower extremity ulcer were excluded in the validation because these outcomes were not routinely sought or recorded in ASCEND. A total of 99% of participants had complete follow-up information, with only 1% of participants lost to follow up and censored. For outcomes included in the analysis, we matched the endpoints as closely as possible to the respective endpoint definitions in the UKPDS-OM2 (see Appendix Table 2 in Supplemental Materials found at https://doi.org/10.1016/j.jval.2021.09.005).

### Statistical Analysis

The UKPDS-OM2 was used to estimate the annual predicted probability of a first occurrence of each endpoint for each patient with T2D in the ASCEND cohort. A total of 10 000 Monte-Carlo replications were used for each patient to minimize stochastic uncertainty. The predicted cumulative incidence of each endpoint from the model was compared with the observed cumulative incidence of the corresponding endpoint in ASCEND accounting for competing risk (from deaths for nonfatal events and from deaths from other causes for cause-specific deaths) and controlling for and excluding effects of allocation to aspirin and omega-3 fatty acids treatments.^26^^,^^27^ The 95% confidence intervals were estimated using 1000 bootstrap samples with replacement.^31^

Performance of the UKPDS-OM2 was assessed based on its calibration (ie, agreement between predicted and observed cumulative incidence) and discrimination (ie, the ability of the model to differentiate individuals who experience events from those who do not).^32^ Calibration was assessed graphically for each endpoint by comparing the year-on-year (up to 10 years) cumulative incidence observed in ASCEND with that predicted by the UKPDS-OM2. The percentage error and the absolute difference between the predicted and observed 7-year cumulative incidence were used to quantify and compare the magnitude of discordance for each endpoint. The mean absolute percentage error (MAPE) over 7 years was used to compare the overall accuracy across endpoints. Calibration was also assessed for each endpoint by dividing the cohort into risk deciles based on 7-year cumulative incidence predicted by the UKPDS-OM2 and plotting the observed 7-year cumulative incidence against that predicted for each decile. We further assessed model calibration in subgroups (sex, age, duration of diabetes, glycated hemoglobin [HbA1c], and body mass index [BMI] at baseline) for mortality outcomes. Uno’s c-statistic estimated at 7 years of follow up was used to assess model’s discrimination of risk for different endpoints.^33^

### Sensitivity Analysis

To assess the impact of using the risk factor progression equations in the UKPDS-OM2 to impute missing risk factor trajectories over time, we considered the alternative scenario whereby all risk factors were held constant at their baseline value over years in the model. That is, if a patient had baseline HbA1c of 6.0%, their HbA1c was assumed to be 6.0% for the entire duration of simulation. Additionally, we excluded participants with missing baseline information (for variables collected in ASCEND) in the ASCEND validation cohort to assess if the results are robust to imputation of baseline information. Finally, to assess the impact of aspirin and omega-3 fatty acid treatment adjustment on the endpoints, we excluded participants who were assigned to receive either one or both treatments in ASCEND and present a comparison of predicted and observed cumulative incidence of each endpoint only among participants allocated to placebo aspirin and placebo omega-3 fatty acid.

All analyses were performed in R 3.5.1.^34^ The reporting of this validation study is in accordance with the TRIPOD checklist^35^ (Appendix Section 2 in Supplemental Materials found at https://doi.org/10.1016/j.jval.2021.09.005).

## Results

Of 15 480 participants in ASCEND, all participants with T2D (N = 14 569 [94%]) formed the validation cohort (Table 1). Participants in this cohort were mostly white (98%). At baseline, they were on average 64 years old, with a median duration of diabetes of 6 years and no previous CVD. They were generally well managed with average HbA1c 7.1% (54 mmol/mol), total cholesterol 4.1 mmol/liter, and systolic blood pressure 136 mm Hg. A summary of the baseline characteristics, after imputing missing data required by the UKPDS-OM2, can be found in Appendix Table 3 in Supplemental Materials found at https://doi.org/10.1016/j.jval.2021.09.005. A comparison of baseline characteristics of participants with T2D in ASCEND and in the UKPDS can be found in Appendix Table 4 in Supplemental Materials found at https://doi.org/10.1016/j.jval.2021.09.005.

**Table 1:** Baseline characteristics of the participants with type 2 diabetes in ASCEND.

Characteristic	Type 2 diabetes cohort in ASCEND∗ (N = 14 569), n (%)	Missing data, n (%)
Male	9166 (63)	0 (0)
Age (years)	63.8 (8.9)	0 (0)
<60	4890 (34)	
60 to <70	6078 (42)	
≥70	3601 (25)	
Ethnicity		43 (<1)
White	14 037 (96)	
South Asian	138 (1)	
Afro-Caribbean	181 (1)	
Other	170 (1)	
Diabetes duration (years)	6 (3-11)	856 (6)
Current smoker	1195 (8)	162 (1)
HbA1c (%)	7.1 (1.2)	5303 (36)
HbA1c (mmol/mol)	54 (13.1)	5303 (36)
Systolic blood pressure (mm Hg)	136.4 (15.2)	4141 (28)
Diastolic blood pressure (mm Hg)	77.3 (9.4)	4148 (28)
BMI (kg/m^2^)	31.4 (6.5)	109 (1)
eGFR (mL/min/1.73 m^2^)	84.5 (20.9)	5304 (36)
Total cholesterol (mmol/liter)	4.1 (0.9)	5300 (36)
HDL cholesterol (mmol/liter)	1.2 (0.3)	5316 (36)
Urinary albumin ≥50 mg/liter	681 (7)	5328 (37)
Diabetes management		0 (0%)
Diet only	2529 (17)	
Any hypoglycemic agent but not insulin	9020 (62)	
Insulin ± other hypoglycemic agent	3020 (21)	
Self-reported hypertension	9141 (63)	105 (1)
Self-reported diabetic retinopathy	2523 (17)	140 (1)
Use of cardiovascular treatments		0† (0)
ACE inhibitor or ARB	8634 (59)	
Beta-blocker	1989 (14)	
Calcium channel blocker	3659 (25)	
Thiazide or related diuretic	2866 (20)	
Statin	11 078 (76)	
NSAID	1280 (9)	
Aspirin (treatment allocation in study)	7282 (50)	

*Note.* Values are mean (SD) or median (interquartile range) for continuous factors and n (%) for categorical factors.

ACE indicates angiotensin converting enzyme; ARB, angiotensin II receptor blocker; ASCEND, A Study of Cardiovascular Events in Diabetes; BMI, body mass index; eGFR, estimated glomerular filtration rate; HbA1c, glycated hemoglobin; HDL, high density lipoprotein; NSAID, nonsteroidal anti-inflammatory drug.

∗ Missing values are excluded from tabulation; percentages are calculated excluding participants with missing information for each variable.

† Concomitant medication use was self-reported.

**Figure 1:**
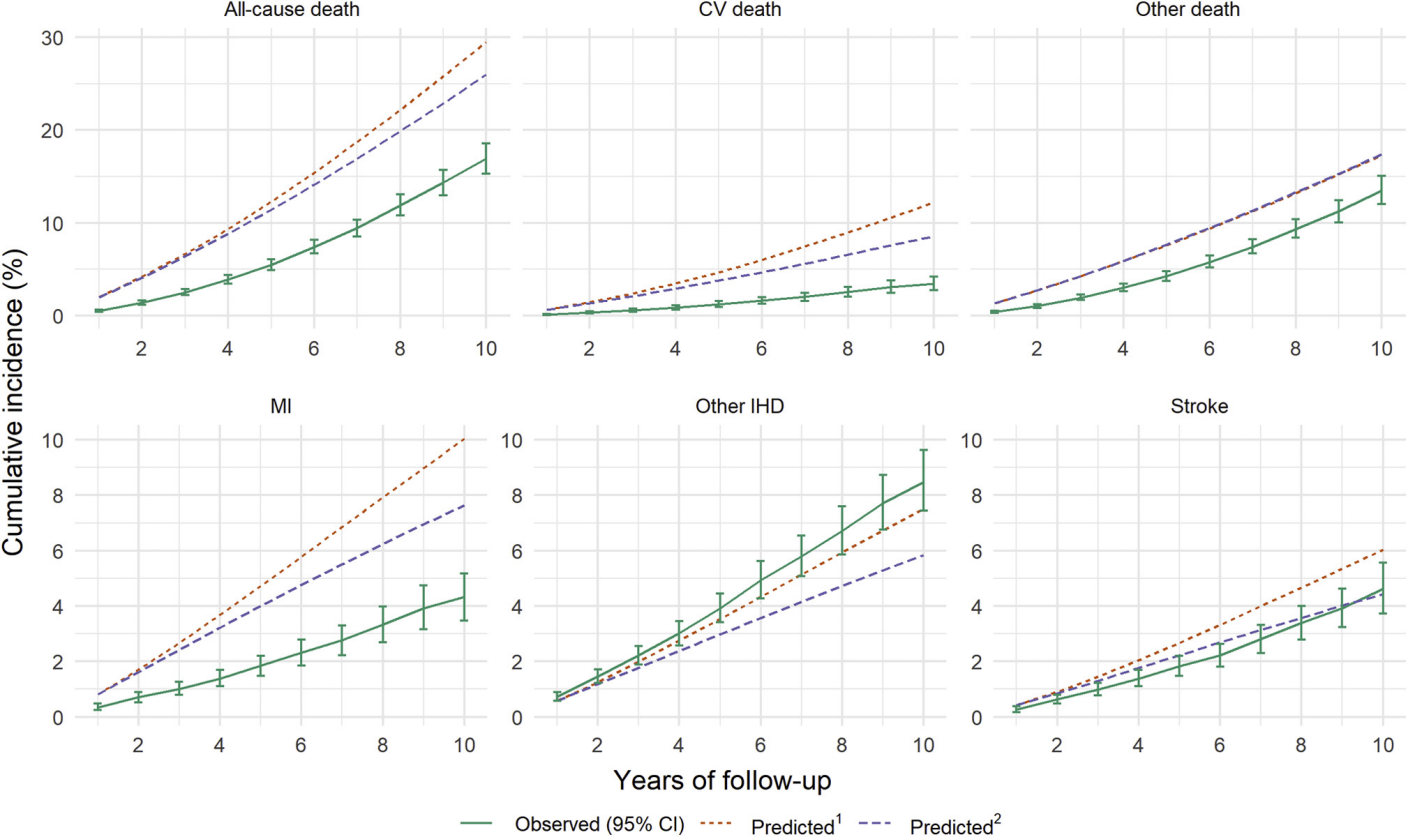
Comparison of cumulative incidence of complications predicted by the UKPDS-OM2 over 10 years with that observed in ASCEND. CV death is defined as death from MI, other IHD, heart failure, or stroke (as in UKPDS-OM2). ^1^UKPDS-OM2 risk factor progression equations used to project risk factor values during follow up (base case). ^2^Values of risk factors during follow up fixed to baseline values (sensitivity analysis).

At 7 years of follow up, cumulative incidence of all-cause death predicted by UKPDS-OM2 was overestimated by 98% (absolute difference 9.3%), which is due to the model overpredicting both CV death by 269% (absolute difference 5.5%) and deaths from other causes by 52% (absolute difference 3.9%) (Fig. 1 and Table 2). The cumulative incidences of MI and stroke were also overpredicted by 149% (absolute difference 4.1%) and 42% (absolute difference 1.2%), respectively. By contrast, the observed cumulative incidence of other IHD was similar to that predicted by the UKPDS-OM2. This was driven by the much higher rate of coronary revascularizations in ASCEND (accounting for 40% of events contributing to the other IHD endpoint) than the UKPDS study period. Excluding coronary revascularization from the other IHD endpoint, we see similar overprediction as for MI (see Appendix Fig. 1 in Supplemental Materials found at https://doi.org/10.1016/j.jval.2021.09.005). For exploratory purposes, we further looked at calibration of the model for heart failure and amputation (see Appendix Table 5 and Appendix Fig. 2 in Supplemental Materials found at https://doi.org/10.1016/j.jval.2021.09.005). Heart failure was overpredicted by 197% (absolute difference, 2.4%). Amputation was well predicted for the first 7 years. Nevertheless, in contrast to the gradual decrease in the rate of amputation observed in ASCEND, the amputation rate was predicted to steadily increase, resulting in the divergence between observed and predicted cumulative incidence in later years. After stratifying by decile of predicted 7-year cumulative incidence, greater deviations were noted between the observed and predicted risks for participants at higher predicted risks for all endpoints, except for other IHD and amputation (see Appendix Fig. 3 in Supplemental Materials found at https://doi.org/10.1016/j.jval.2021.09.005). Overall, over 7 years of follow up, the MAPE of the UKPDS-OM2 was worst for CV death (302%) and heart failure (283%).

CI indicates confidence interval; CV, cardiovascular; IHD, ischemic heart disease; MI, myocardial infarction; UKPDS-OM2, UK Prospective Diabetes Study Outcomes Model 2.

**Table 2:** Comparison of cumulative incidence of complications predicted by the UKPDS-OM2 with observed rates at 7 years of follow up for participants in ASCEND.

Endpoints	Observed cumulative incidence, % (95% CI)	Predicted∗				Predicted†			
		Cumulative incidence, %	% error	MAPE, %	c-statistic	Cumulative incidence, %	% error	MAPE, %	c-statistic
All-cause death	9.4 (8.5-10.3)	18.7	98	158	0.72	17.0	79	146	0.73
CV death	2.0 (1.6-2.5)	7.5	269	302	0.70	5.6	178	246	0.71
Other death	7.4 (6.6-8.2)	11.3	52	115	0.72	11.3	53	116	0.73
MI	2.8 (2.3-3.3)	6.9	149	151	0.58	5.5	100	122	0.58
Other IHD	5.8 (5.1-6.5)	5.1	−12	13	0.60	4.2	−28	23	0.60
Stroke	2.8 (2.3-3.3)	4.0	42	47	0.66	3.1	12	29	0.66

*Note.* % error defined as percentage error; CV death is defined as death from MI, other IHD, heart failure, or stroke (as in UKPDS-OM2). % error is the degree of overprediction as percentage of observed (negative values represent underprediction) calculated using the cumulative incidences at year 7. MAPE is the absolute percentage error averaged across the 7 years of follow-up.

ASCEND indicates A Study of Cardiovascular Events in Diabetes; CI, confidence interval; CV, cardiovascular; IHD, ischemic heart disease; MAPE, mean absolute percentage error; MI, myocardial infarction; UKPDS-OM2, UK Prospective Diabetes Study Outcomes Model 2.

∗ UKPDS-OM2 risk factor progression equations used to project risk factor values during follow-up (base case).

† Values of risk factors during follow-up fixed to baseline values (sensitivity analysis).

Discrimination of the model for MI and other IHD (c-statistics of 0.58 and 0.60, respectively) was poorer than that for other endpoints (c-statistics ranging from 0.66 to 0.75) (Table 2 and see Appendix Table 5 in Supplemental Materials found at https://doi.org/10.1016/j.jval.2021.09.005). This is also reflected in the plot comparing predicted with observed 7-year cumulative incidence stratified by predicted risk (see Appendix Fig. 3 in Supplemental Materials found at https://doi.org/10.1016/j.jval.2021.09.005), where although clear gradients of increasing risks were observed across deciles of predicted risk for other endpoints, these gradients were much less distinct for MI and other IHD across the risk deciles.

Overall, the model overpredicted mortality across all risk factor subgroups explored (sex, age, HbA1c, duration of diabetes, BMI) (see Appendix Figs. 4-8 in Supplemental Materials found at https://doi.org/10.1016/j.jval.2021.09.005). Lower levels of bias were observed in the following subgroups (see Appendix Table 6 in Supplemental Materials found at https://doi.org/10.1016/j.jval.2021.09.005): for CV deaths, participants below 60 years old (MAPE 99% compared with 350% for participants between 60 and 70 years old and 403% for participants at least 70 years old) and participants with a duration of diabetes of at least 5 years (MAPE 256% for participants with >10 years of diabetes, 197% for participants with 5-10 years of diabetes, and 658% for participants with <5 years of diabetes), and for death from other causes, participants with HbA1c of at least 7.5% (MAPE 97% compared with 192% for participants with HbA1c <7.5%) and participants with BMI of at least 35 kg/m^2^ (MAPE 24%, compared with at least 100% for participants with BMI <35 kg/m^2^).

### Sensitivity Analyses

In the sensitivity analyses in which all risk factors were held constant at their baseline values, the predicted cumulative incidences were smaller than in the main analyses, with somewhat smaller discrepancies than the observed cumulative incidences. Nevertheless, the model still substantially overpredicted death, MI, and heart failure rates (percentage error at year 7, 79%, 100%, and 134%, respectively). Amputation is well predicted over 10 years.

In the subgroup of participants with complete baseline information (n = 7578), with the exception of heart failure, the percentage error at year 7 and the MAPE across 7 years are both higher in this subgroup of participants across all endpoints, particularly for CV death and MI (see Appendix Table 7 and Appendix Fig. 9 in Supplemental Materials found at https://doi.org/10.1016/j.jval.2021.09.005). Discrimination and absolute differences between the predicted and observed cumulative incidences across all endpoints were similar to results in the main analyses with missing data imputed. In the subgroup of participants with allocated placebo aspirin and placebo omega-3 fatty acid (n = 3651), the performance of the UKPDS-OM2 is similar to the main analyses with adjustments for effects of allocation to aspirin and omega-3 fatty acids treatments (see Appendix Table 8 and Appendix Fig. 10 in Supplemental Materials found at https://doi.org/10.1016/j.jval.2021.09.005).

## Discussion

The UKPDS-OM2 substantially overpredicted the risks of MI, heart failure, and death (particularly CV death) over 10 years of follow up in ASCEND. Stroke was also overpredicted, although to a lesser extent. In terms of discrimination, the model was positively predictive for all endpoints, but discriminated risks of MI and other IHD less well than other endpoints.

Despite MI being overpredicted, other IHD (which includes coronary revascularizations) appears well predicted. Nevertheless, the number of coronary revascularizations performed each year in the United Kingdom has increased over the last decades^36^ with percutaneous coronary interventions increasing 3-fold between 2000 and 2010. Patients are now more likely to receive preventive coronary revascularization possibly contributing to the decline in the risk of MI and the apparent increase in the risk of other IHD (which includes coronary revascularization). Excluding coronary revascularization from the other IHD endpoint leads to a similar pattern of overprediction (see Appendix Fig. 1 in Supplemental Materials found at https://doi.org/10.1016/j.jval.2021.09.005). Furthermore, the observed cumulative incidence of a combined endpoint of MI or coronary revascularization (assuming that coronary revascularizations prevented some MIs) was also lower than the cumulative incidence of MI predicted by the UKPDS-OM2. These exploratory analyses suggest that the UKPDS-OM2 is overpredicting the risk of IHD in general and that other IHD seems well predicted partly because of the higher rate of coronary revascularizations in ASCEND.

The UKPDS is a significantly older trial in which participants were followed between 1977 and 2007. Compared with UKPDS participants, ASCEND participants had better managed lipids (75% of ASCEND participants took a statin vs 0.3% on lipid-lowering drugs in the UKPDS), blood pressure (67% of ASCEND participants on antihypertensive drugs vs 12% in the UKPDS), and blood glucose (similar HbA1c levels despite ASCEND participants having an average of 6 years of diabetes vs newly diagnosed in UKPDS); higher use of coronary revascularization; and reduced likelihood of smoking (8% in ASCEND vs 31% in UKPDS) (see Appendix Table 4 in Supplemental Materials found at https://doi.org/10.1016/j.jval.2021.09.005).^37^ This may reflect earlier diagnosis of diabetes in ASCEND participants compared with participants in UKPDS and improved risk factor control in recent years. Given that the overprediction for death and MI is consistently observed in other validation studies,^17^, ^18^, ^19^, ^20^ this suggest that perhaps improvements in diabetes and CVD diagnosis and treatment and dietary and lifestyle changes have an impact on CV risks beyond the impact from risk factors accounted for by the UKPDS-OM2. Previous studies have also observed a decline in age-adjusted CVD incidence and case-fatality rates in the last 2 decades,^36^^,^^38^, ^39^, ^40^, ^41^ over and above the effects explained by improvement in risk factors.^42^, ^43^, ^44^, ^45^ Therefore, the UKPDS-OM2 requires calibration or de novo estimation of at least mortality and CV event risks using contemporary patient data.

Additionally, the performance of the UKPDS-OM2 was found to be poorer in patients who were older or had a shorter duration of diabetes at baseline. In particular, poorer performance in older patients was also observed in a Dutch and an Italian cohort from an earlier patient-level validation study.^20^ In the UKPDS, participants received a diagnosis of diabetes at a mean age of 53 years.^37^ In contrast, patients in ASCEND (mean age 64; mean duration of diabetes 6), the Dutch cohort (mean age 65, mean duration of diabetes 7), and the Italian cohort (mean age 68; mean duration of diabetes 11) were diagnosed on average approximately 5 years later. Diagnosis of diabetes at an older age compared with a younger age has been found to be associated with a slower rate of glycemic deterioration^46^ and lower CV and mortality risks.^47^ The UKPDS data may not generalize well to older patients who received a diagnosis of diabetes at an older age, which may explain the poorer performance of the model in this patient category. Hence, new equations based on more contemporary cohorts would be helpful in better capturing disease risks in these patient categories.

The 2 major strengths of the present analysis are the large sample size of ASCEND and its high-quality individual patient data, with complete follow-up data for 99% of participants and adjudicated endpoints (for MI, other IHD, stroke, and deaths). The model validation was also performed on a UK patient cohort, which eliminates the uncertainty because of geographic and ethnic differences present in previous external validations of the UKPDS-OM2 that were performed in North American or other European cohorts.^17^, ^18^, ^19^

Limitations of the analyses should also be acknowledged. First, data on some baseline risk factors such as low-density lipoprotein cholesterol, hemoglobin, and white blood cell count were not available in ASCEND and were imputed based on their relationships with other markers (eg, total cholesterol) derived in external data sets. This assumes the generalizability of such relationships to participants in the ASCEND cohort. For other risk factors with missing data, the missing at random assumption was made in imputing these values. In the subset of participants with no missing baseline data (for variables collected in ASCEND), similar magnitudes of overprediction and discrimination were found. MAPE of the UKPDS-OM2 was generally higher suggesting that the bias reported may be an underestimation, but our conclusion is still robust. Second, follow-up blood and urine samples were only collected in a random sample of about 1200 ASCEND participants, so risk factors were projected over time using the risk factor progression equations in the UKPDS-OM2. Exploratory tabulations (see Appendix Fig. 11 in Supplemental Materials found at https://doi.org/10.1016/j.jval.2021.09.005) suggest that the rate of deterioration of these cardiometabolic markers was slightly overpredicted at 2.5 years into the follow up. We do not have sufficient data to reliably assess the validity of these equations for the ASCEND participants. Nevertheless, the use of these equations to project missing risk factor progression over time reflects how other users are likely to use the model in absence of risk factor progression data. Moreover, we present results with risk factors held constant at their baseline values to provide a benchmark for the range within which the actual predictions could lie. Third, although adverse events in ASCEND were chosen to match the definitions of endpoints in the UKPDS-OM2, we acknowledge that differences may exist. Nevertheless, these are unlikely to materially affect the degree of discrepancy in event rates reported here. Additionally, most microvascular endpoints in the UKPDS-OM2 (blindness, end-stage renal failure, and lower extremity ulcer) were excluded from the analysis because they were not routinely sought or recorded in ASCEND and incidence of occurrence of these endpoints could not be reliably estimated. Fourth, the inclusion/exclusion criteria in ASCEND may restrict the generalizability of results to other T2D cohorts. Participants in ASCEND had no previous CVD (exclusion criteria in ASCEND) despite a median duration of diabetes of 6 years,^48^ in contrast to the 6% and 4% of participants with MI and other IHD, respectively, 6 years after diabetes diagnosis in the UKPDS.^10^ There are also further exclusion criteria in ASCEND (eg, patients with liver disease and blindness excluded) that make the ASCEND cohort more selective. Additionally, participants in ASCEND were predominantly white. Nevertheless, many of these factors are covariates in the UKPDS-OM2 risk equations, and it is expected that the model is able to account for these differences. There may be other systematic differences between the ASCEND and the UKPDS cohorts that we do not observe and are unaccounted for, but these differences are unlikely to account for the degree of discrepancy in event rates reported here. Nevertheless, caution needs to be taken when generalizing these results to other T2D cohorts.

Previous validation studies have demonstrated that, despite overestimating absolute risks, models can predict the relative risks of treatment well.^49^ Although estimation of relative effects is important, accurate estimates of absolute effects are necessary if the aim is to evaluate the magnitude of impact of the treatment on number of adverse events, costs, and (quality-adjusted) life-years. Overestimating disease risks will directly result in overestimating health benefit, which is likely to affect cost-effectiveness.^50^^,^^51^ As an illustration, for the ASCEND T2D cohort included in this study, a 15% risk reduction in CV death results in 0.058 increase in quality-adjusted life-years (QALYs) based on the 7-year CV death risk observed in ASCEND, in contrast to 0.218 QALYs based on the overestimated 7-year CV death risk predicted by the UKPDS-OM2 (see Appendix Table 9 in Supplemental Materials found at https://doi.org/10.1016/j.jval.2021.09.005). The difference in incremental lifetime cost is less pronounced (£6137 vs £5579), which overall translates into substantial difference in the incremental cost-effectiveness ratio (£105 540 per QALY vs £25 585 per QALY). Furthermore, the magnitude of bias in these incremental impacts can vary across different risk groups, as illustrated by the separate comparisons among participants in the lowest and highest CV death risk deciles (see Appendix Table 9 and Appendix Section 3 in Supplemental Materials found at https://doi.org/10.1016/j.jval.2021.09.005).

The results from our analyses, together with findings from previous external validations of the UKPDS-OM2,^17^, ^18^, ^19^ indicate that the UKPDS-OM2 may not provide reliable estimates of absolute event rates for contemporary T2D cohorts such as ASCEND. The model would need to be recalibrated or new risk equations need to be estimated to predict long-term outcomes in contemporary T2D cohorts.

## Conclusions

To confidently use a prediction model, its ability to reliably predict outcomes in cohorts of patients beyond those used in model development needs to be confirmed. In this study, the UKPDS-OM2 substantially overpredicted risks of CV death, other death, and MI in a large cohort of patients with T2D. Appropriate adjustments would be needed when using the model for clinical or economic analyses in contemporary cohorts such as ASCEND particularly when absolute risk or total cost and (quality-adjusted) life expectancy is of interest, or perhaps new models for predicting long-term outcomes in T2D may be required.

## Article and Author Information

**Author Contributions:***Concept and design:* Keng, Leal, Mihaylova

*Acquisition of data:* Mafham, Bowman, Armitage

*Analysis and interpretation of data:* Keng, Leal, Mihaylova

*Drafting of the manuscript:* Keng

*Critical revision of the paper for important intellectual content:* Keng, Leal, Mafham, Armitage, Mihaylova

*Statistical analysis:* Keng, Leal, Mihaylova

*Provision of study materials or patients:* Mafham, Bowman, Armitage

*Obtainingfunding:* Bowman, Armitage, Mihaylova

*Supervision:* Leal, Bowman, Armitage, Mihaylova

**Conflict of Interest Disclosures:** Ms Keng reported receiving a DPhil stipend from the British Heart Foundation Centre of Research Excellence, Oxford, England, United Kingdom (grant code: RE/13/1/30181). Dr Leal reported a research grant paid to his institution from Innovative Medicines Initiative 2 Joint Undertaking under grant agreement number 115 881 (RHAPSODY). Dr Mafham reported her institution receiving research grants from the British Heart Foundation; reported core departmental funding from the Medical Research Council and Cancer Research UK paid to her institution; reported that Bayer Pharma AG provided the aspirin and placebo and funding for drug packaging to her institution and Solvay, Abbott, and Mylan provided the omega-3 and placebo and funding for drug packaging to her institution; reported a grant paid to her institution from The Medicines Company/Novartis; and reported being a board member (unpaid) of NHS DigiTrials, a member (unpaid) of the steering committees for ORION-4 trial, ASCEND trial, RECOVERY trial, and a member (unpaid) of the funding committee for Kidney Research UK. Dr Bowman reported receiving grants paid to her institution from the British Heart Foundation, Medical Research Council, Cancer Research UK, Novartis, and the National Institute for Health Research Oxford Biomedical Research Centre; reported Bayer Pharma AG provided the study aspirin and placebo and funding for drug packaging; and reported that Solvay, Abbott, and Mylan provided the study omega-3 and placebo and funding for drug packaging. Dr Armitage reported grants paid to Oxford University for the trial from British Heart Foundation; reported core grants paid to Oxford University from the Medical Research Council and Cancer Research UK; reported grants, aspirin, and placebo tablets from Bayer; reported grants, fish oil capsules, and placebo from Mylan, Abbott, and Solvay; reported grants paid to Oxford University for the trial from The Medicines Company and Novartis; reported a grant paid to Oxford University from the National Institute for Health Research Health Technology Assessment; reported reimbursement for travel costs from Bayer; and reported being a participant (unpaid) on a data safety monitoring board or advisory board. Dr Mihaylova reported grants paid to the University of Oxford from the National Institute for Health Research Oxford Biomedical Research Centre and is an editor of *Value in Health* and had no role in the peer review process of this article.

**Funding/Support:** This study was supported by a grant from British Heart Foundation Centre of Research Excellence, Oxford, England, United Kingdom (grant code: RE/13/1/30181).

**Role of the Funder/Sponsor:** The funder had no role in the design and conduct of the study; collection, management, analysis, and interpretation of the data; preparation, review, or approval of the manuscript; and decision to submit the manuscript for publication.
