# Supplementary material for: Performance of the UK Prospective Diabetes Study Outcomes Model 2 in a Contemporary UK Type 2 Diabetes Trial Cohort
Source: Value Health. 2022 Mar;25(3):435–42. doi: 10.1016/j.jval.2021.09.005 (PMC8881217; doi:10.1016/j.jval.2021.09.005)
Supplement: Appendix [file mmc1.docx]

Performance of the UKPDS Outcomes Model 2 in a contemporary UK type 2 diabetes trial cohort

Supplementary materials

**Table of Contents**

[Appendix Tables 3](#_Toc85555128)

[Appendix Table 1: Inputs required for UKPDS-OM2 and their availability in ASCEND 3](#_Toc85555129)

[Appendix Table 2: Definitions of outcomes in UKPDS-OM2 and identifiers used to identify corresponding adverse events in ASCEND 4](#_Toc85555130)

[Appendix Table 3: Baseline characteristics of participants with type 2 diabetes in ASCEND required by UKPDS-OM2, before and after imputation 6](#_Toc85555131)

[Appendix Table 4: Comparison of baseline characteristics of participants with type 2 diabetes in ASCEND and in the UKPDS 7](#_Toc85555132)

[Appendix Table 5: Performance of UKPDS-OM2 for heart failure and amputation at year 7 for participants in ASCEND validation cohort 8](#_Toc85555133)

[Appendix Table 6: Mean absolute prediction error [MAPE (%)] from comparison of cumulative incidence predicted by the UKPDS-OM2 and observed for participants in the ASCEND validation cohort over 7 years, by risk factor subgroups 9](#_Toc85555134)

[Appendix Table 7: Performance of UKPDS-OM2 at year 7 for participants in ASCEND validation cohort, for participants with complete baseline information (n = 7578) 10](#_Toc85555135)

[Appendix Table 8: Performance of UKPDS-OM2 at year 7 for participants in ASCEND validation cohort, for participants in placebo aspirin and placebo omega-3 fatty acid arm only (n = 3651) 11](#_Toc85555136)

[Appendix Table 9: Predicted lifetime cost, life expectancy and quality-adjusted life years from 15% risk reduction in cardiovascular death, based on risk predicted by the UKPDS-OM2 and observed in ASCEND 12](#_Toc85555137)

[Appendix Figures 13](#_Toc85555138)

[Appendix Figure 1: Comparison of cumulative incidence of MI and Other IHD, predicted by the UKPDS-OM2 over 10 years with that observed in ASCEND, including and excluding coronary revascularizations 13](#_Toc85555139)

[Appendix Figure 2: Comparison of cumulative incidence of heart failure and amputation predicted by the UKPDS-OM2 over 10 years with that observed in ASCEND 14](#_Toc85555140)

[Appendix Figure 3: Comparison of 7-year cumulative incidence of complications predicted by the UKPDS-OM2 with that observed in ASCEND, stratified by decile of predicted 7-year cumulative incidence for each outcome 15](#_Toc85555141)

[Appendix Figure 4: Comparison of cumulative incidence of complications predicted by the UKPDS-OM2 over 10 years with that observed in ASCEND, by sex 16](#_Toc85555142)

[Appendix Figure 5: Comparison of cumulative incidence of complications predicted by the UKPDS-OM2 over 10 years with that observed in ASCEND, by age at baseline 17](#_Toc85555143)

[Appendix Figure 6: Comparison of cumulative incidence of complications predicted by the UKPDS-OM2 over 10 years with that observed in ASCEND, by HbA1c at baseline 18](#_Toc85555144)

[Appendix Figure 7: Comparison of cumulative incidence of complications predicted by the UKPDS-OM2 over 10 years with that observed in ASCEND, by duration of diabetes at baseline 19](#_Toc85555145)

[Appendix Figure 8: Comparison of cumulative incidence of complications predicted by the UKPDS-OM2 over 10 years with that observed in ASCEND, by BMI at baseline 20](#_Toc85555146)

[Appendix Figure 9: Comparison of cumulative incidence of complications predicted by the UKPDS-OM2 over 10 years with that observed in ASCEND, for participants with complete baseline information (N = 7578) 21](#_Toc85555147)

[Appendix Figure 10: Comparison of cumulative incidence of complications predicted by the UKPDS-OM2 over 10 years with that observed in ASCEND, for participants in aspirin placebo and omega-3 placebo arm only (N = 3651) 22](#_Toc85555148)

[Appendix Figure 11: Average values of risk factors predicted by the UKPDS risk factor equations (solid black line) compared to those observed (marked ‘X’) in a random sample of ASCEND participants^†^ 24](#_Toc85555149)

[Appendix Section 1: Missing baseline data 25](#_Toc85555150)

[Appendix Section 2: TRIPOD statement 27](#_Toc85555151)

[Appendix Section 3: Estimating impact of over-predicting CV death risk on cost-effectiveness 29](#_Toc85555152)

#

# Appendix Tables

## Appendix Table 1: Inputs required for UKPDS-OM2 and their availability in ASCEND

| Inputs required for UKPDS-OM2 | n (%) missing in ASCEND | Notes |
| --- | --- | --- |
| **Baseline characteristics** |  |  |
| Ethnicity (White, South Asian, Afro-Carribean) | 43 (0.3) | Categories in ASCEND: White, Indian/Pakistani/Bangladeshi (South Asian), African/Caribbean (Afro-Carribean), Other, unknown  Participants with other/unknown ethnicity were grouped together with White. |
| Gender | 0 (0) |  |
| Age | 0 (0) |  |
| Duration of diabetes | 856 (5.9) |  |
| Weight (kg) | 84 (0.6) |  |
| Height (m) | 53 (0.4) |  |
| Atrial fibrillation (Y/N; defined from Minnesota codes 831 [atrial fibrillation (persistent)] and 833 [intermittent atrial fibrillation]) | ✝ | Assume no atrial fibrillation – patients on anti-coagulants excluded at baseline |
| Peripheral vascular disease (Y/N; defined from presence of intermittent claudication or ankle brachial pressure index < 0.9) | ✝ | Assume no peripheral vascular disease |
| Current smoker (Y/N) | 162 (1.1) |  |
| Albuminuria (Y/N; defined as presence of micro-/macro-albuminuria, urinary albumin $\geq$ 50mg/L) | 5328 (36.6) | Albuminuria defined as Urinary albumin $\geq$ 50mg/l . |
| **Biomarkers** |  |  |
| HDL cholesterol (mmol/L; CDC aligned assay) | 5316 (36.5) |  |
| LDL cholesterol (mmol/L; CDC aligned assay) | ✝ | Triglycerides not measured in ASCEND. Predicted LDL from Apo(B), HDL cholesterol, non-HDL cholesterol and other biomarkers. Further details in Supplementary Section 1. |
| Systolic blood pressure (mm Hg) | 4141 (28.4) |  |
| HbA1c (%; DCCT/NGSP aligned assay) | 5303 (36.4) |  |
| Heart rate (bpm) | 5020 (34.5) |  |
| White blood count (x ${10}^{9}$ per litre) | ✝ |  |
| Haemoglobin (g/dL) | ✝ |  |
| eGFR (mL/min/1.73m^2^; estimated from serum creatinine using MDRD equation) | 5304 (36.4) | In ASCEND, eGFR estimated from blood cystatin C levels using CKD-EPI equation |
| **History of complication (Y/N)** |  |  |
| Myocardial infarction | ǂ | No history of myocardial infarction |
| Stroke | ǂ | No history of stroke |
| Other ischaemic heart disease | ǂ | No history of other ischaemic heart disease |
| Heart failure | ǂ | No history of heart failure |
| Blindness | § | Assume no history of blindness |
| Renal failure | § | Assume no history of renal failure |
| Ulcer | § | Assume no history of ulcer |

HbA1c, glycated haemoglobin; eGFR, estimated glomerular filtration rate; HDL, high density lipoprotein; Apo(B), apolipoprotein B.

✝: Not available in ASCEND; ǂ: In ASCEND, patients with previous cardiovascular disease were excluded from the trial; §: Participants were assumed not to have (and likely did not have, as individuals with compliance limiting conditions were ineligible) a history of blindness, renal failure and ulcer at baseline.

## Appendix Table 2: Definitions of outcomes in UKPDS-OM2 and identifiers used to identify corresponding adverse events in ASCEND

| Outcomes | Definition in UKPDS-OM2 (ICD-9 codes)^1,2^ | Outcomes in ASCEND^3^ |
| --- | --- | --- |
| All-cause death | All-cause death | All-cause death |
| Cardiovascular death | Deaths from myocardial infarction, other ischaemic heart disease, stroke, and heart failure as defined below | Deaths from myocardial infarction, other ischaemic heart disease, stroke, and heart failure as defined below |
| Other death | All-cause death excluding cardiovascular death (as defined in the UKPDS) | All-cause death excluding cardiovascular death (as defined in ASCEND) |
| Myocardial infarction | WHO clinical criteria with electrocardiogram/enzyme changes or new pathological Q wave  410 (Acute myocardial infarction);  ≥ 798 & ≤ 798.9 (Sudden death) | Myocardial infarction (fatal/non-fatal)  “Evidence of cardiac necrosis (consistent elevation in cardiac biomarkers or relevant autopsy findings) and there was other evidence of an acute MI (including symptoms of ischemia, recent coronary intervention, death, new ECG changes, evidence of a new myocardial defect on cardiac imaging or an acute coronary occlusion at angiography) and no other diagnosis was likely.” |
| Other ischaemic heart disease | Angina/ischaemic heart disease - WHO clinical criteria confirmed by a new ECG abnormality or an ECG which becomes abnormal on exercise  ≥ 411 & ≤ 414.9 (Ischaemic heart disease excluding acute myocardial infarction) | Angina;  Coronary revascularizations (coronary artery bypass graft, percutaneous transluminal coronary angioplasty);  Death from other coronary heart disease (not myocardial infarction) |
| Stroke | Stroke with symptoms that persisted for more than one month  ≥ 430 & ≤ 434.9 (haemorrhagic, ischemic strokes [for fatal and non-fatal strokes]);  436 (acute but ill-defined strokes [for other non-fatal strokes]) | Any stroke (fatal/non-fatal)  “Acute symptomatic episode of focal or global neurological dysfunction caused by brain, spinal or retinal vascular injury as a result of hemorrhage or infarction which lasted >24 hours, lead to death or was associated with evidence of an acute infarct or hemorrhage on brain imaging corresponding with the clinical syndrome.” |
| Heart failure | ≥ 428 & ≤ 428.9 (Heart failure) | Heart failure (fatal/non-fatal; includes pulmonary oedema) |
| Amputation | Amputation of digit or limb for any reason  Procedure codes ≥ 5.845 & ≤ 5.848 (Amputation of digit or limb);  250.6 (diabetes with peripheral circulatory disorder);  Fatal peripheral vascular event  997.2 (peripheral vascular complications);  997.6 (late amputation stump complication);  250.6 (diabetes with peripheral circulatory disorder);  440.2 (atherosclerosis of arteries of extremities) | Amputation (above knee, toe, below knee, foot, leg, finger/thumb) |

**References**

1. UK Prospective Diabetes Study Group. UK Prospective Diabetes Study (UKPDS). VIII. Study design, progress and performance. *Diabetologia* 1991; 34: 877–890.
2. Hayes AJ, Leal J, Gray AM, et al. UKPDS Outcomes Model 2: a new version of a model to simulate lifetime health outcomes of patients with type 2 diabetes mellitus using data from the 30 year United Kingdom Prospective Diabetes Study: UKPDS 82. *Diabetologia* 2013; 56: 1925–1933.
3. The ASCEND Study Collaborative Group. Effects of Aspirin for Primary Prevention in Persons with Diabetes Mellitus. *N Engl J Med* 2018; 379: 1529–1539.

## Appendix Table 3**: Baseline characteristics of participants with type 2 diabetes in ASCEND required by UKPDS-OM2, before and after imputation**

| Validation cohort (N = 14 569) | Before imputation^†^ | After imputation |
| --- | --- | --- |
| **Baseline characteristics** |  |  |
| Male | 9,166 (63%) | 9,166 (63%) |
| Age | 63.8 (8.9) | 63.8 (8.9) |
| Ethnicity |  |  |
| White | 14,037 (98%) | 14,250 (98%) |
| South Asian | 138 (1%) | 138 (1%) |
| Afro-Carribean | 181 (1%) | 181 (1%) |
| Others/Unknown | 213 (2%) | 0 (0%) |
| Duration of diabetes | 6 (3 - 11) | 6 (3-11) |
| Current smoker | 1,195 (8%) | 1,195 (8%) |
| Albuminuria | 681 (7%) | 683 (5%) |
| Atrial fibrillation | NA | 0 (0%) |
| Peripheral vascular disease | NA | 0 (0%) |
| **Biomarkers** |  |  |
| HbA1c (%) | 7.1 (1.2) | 7.1 (1.0) |
| Systolic blood pressure (mm Hg) | 136.4 (15.2) | 136.2 (13.1) |
| BMI (kg/m^2^) | 31.4 (6.5) | 31.4 (6.5) |
| eGFR (mL/min/1.73m^2^) | 84.5 (20.9) | 85.4 (18.3) |
| HDL cholesterol (mmol/L) | 1.2 (0.3) | 1.2 (0.3) |
| LDL cholesterol (mmol/L) | NA | 2.2 (0.5) |
| White blood cell count (x ${10}^{9}$ per litre) | NA | 6.4 (0.6) |
| Haemoglobin (g/dl) | NA | 14.3 (0.8) |
| Heart rate (bpm) | 75.0 (11.5) | 75.1 (9.5) |
| **History of complication** |  |  |
| Myocardial infarction | 0 (0%) | 0 (0%) |
| Stroke | 0 (0%) | 0 (0%) |
| Other ischaemic heart disease | 0 (0%) | 0 (0%) |
| Heart failure | 0 (0%) | 0 (0%) |
| Blindness | NA | 0 (0%) |
| Renal failure | NA | 0 (0%) |
| Ulcer | NA | 0 (0%) |

HbA1c, glycated haemoglobin; BMI, body mass index; eGFR, estimated glomerular filtration rate; HDL, high density lipoprotein; NA, Data not available in ASCEND.

Values are mean (SD) or median (interquartile range) for continuous variables, and N (%) for categorical variables.

^†^Assumptions and further details on method for imputing missing data can be found in Appendix Table 1 and Appendix Section 1.

## Appendix Table 4: Comparison of baseline characteristics of participants with type 2 diabetes in ASCEND and in the UKPDS

|  | Type 2 diabetes cohort in ASCEND^†^  (N = 14,569) | UKPDS^‡^  (N = 3867) |
| --- | --- | --- |
| Male | 63% | 61% |
| Age (years) | 63.8 (8.9) | 53.3 (8.6) |
| Ethnicity |  |  |
| White | 96% | 81% |
| South Asian | 1% | 10% |
| Afro-Carribean | 1% | 8% |
| Other | 1% | 1% |
| Diabetes duration (years) | 6 (3 - 11) | 0 |
| Current smoker | 8% | 31% |
| HbA1c (%) | 7.1 (1.2) | 7.1 (1.5) |
| HbA1c (mmol/mol) | 54 (13.1) | 54 (16.5) |
| Systolic blood pressure (mm Hg) | 136.4 (15.2) | 135 (20) |
| Diastolic blood pressure (mm Hg) | 77.3 (9.4) | 82 (10) |
| BMI (kg/m^2^) | 31.4 (6.5) | 27.5 (5.2) |
| Total cholesterol (mmol/L) | 4.1 (0.9) | 5.4 (1.1) |
| HDL cholesterol (mmol/L) | 1.2 (0.3) | 1.1 (0.2) |
| Diabetes management |  | - |
| Diet only | 2,529 (17%) |  |
| Any hypoglycaemic agent but not insulin | 9,020 (62%) |  |
| Insulin +/- other hypoglycaemic agent | 3,020 (21%) |  |
| Diabetic retinopathy | 17%  (self-reported) | 36% |
| Use of cardiovascular treatments |  |  |
| Antihypertensive | 67%  (ACE inhibitor or ARB; Beta-blocker; Calcium channel blocker) | 12% |
| Lipid lowering | 76%  (statin) | 0.3% |
| Diuretic | 20% | 13% |
| Aspirin | 50%  (treatment allocation in study) | 1.6%  (more than one aspirin daily) |
| Use of diabetes treatments |  |  |
| Insulin | 21% | 45% |
| Sulphonylurea | 28% | 43% |
| Metformin | 69% | 8.8% |
| Other hypoglycaemic treatments | 4.8% | - |

Values are mean (SD) or median (interquartile range) for continuous factors, and % for categorical factors.

^†^Missing values are excluded from tabulation; percentages are calculated excluding participants with missing information for each variable.

^‡^Baseline characteristics of participants in the UKPDS study on intensive blood-glucose control compared with conventional treatment.^1^ Tabulation of diabetes treatment in the UKPDS based on treatment allocation.

**Reference**

1. UK Prospective Diabetes Study (UKPDS) Group. Intensive blood-glucose control with sulphonylureas or insulin compared with conventional treatment and risk of complications in patients with type 2 diabetes (UKPDS 33). The Lancet. 1998 Sep 12;352(9131):837-53.

## Appendix Table 5: Performance of UKPDS-OM2 for heart failure and amputation at year 7 for participants in ASCEND validation cohort

|  | Observed cumulative incidence (%)  (95% CI) | Predicted^1^ | | | |  | Predicted^2^ | | | |
| --- | --- | --- | --- | --- | --- | --- | --- | --- | --- | --- |
| **Endpoints** |  | **Cumulative incidence (%)** | **% error** | **MAPE (%)** | **C-statistic** |  | **Cumulative incidence (%)** | **% error** | **MAPE (%)** | **C-statistic** |
| Heart failure | 1.2 (0.9, 1.6) | 3.6 | 197 | 283 | 0.71 |  | 2.9 | 134 | 246 | 0.71 |
| Amputation | 0.8 (0.5, 1.1) | 1.0 | 26 | 13 | 0.75 |  | 0.6 | -22 | 25 | 0.75 |

CI, confidence interval; % error, percentage error; MAPE, mean absolute percentage error. Percentage error is the degree of over-prediction as percentage of observed (negative values represent under-prediction) calculated using the cumulative incidences at year 7. MAPE is the absolute percentage error averaged across the 7 years of follow-up.

^1^UKPDS-OM2 risk factor progression equations used to project risk factor values during follow-up (base case).

^2^Values of risk factors during follow-up fixed to baseline values (sensitivity analysis).

## Appendix Table 6: Mean absolute prediction error [MAPE (%)] from comparison of cumulative incidence predicted by the UKPDS-OM2 and observed for participants in the ASCEND validation cohort over 7 years, by risk factor subgroups

| Subgroup | All-cause death | CV death | Other death |
| --- | --- | --- | --- |
| By sex |  |  |  |
| Male | 157 | 260 | 120 |
| Female | 165 | 450 | 108 |
| By age |  |  |  |
| <60 | 92 | 99 | 85 |
| >=60 <70 | 159 | 350 | 103 |
| >=70 | 181 | 403 | 130 |
| By HbA1c (%) |  |  |  |
| <7.5 | 248 | 466 | 192 |
| >=7.5 | 155 | 412 | 97 |
| By duration of diabetes (years) | |  |  |
| <5 | 192 | 658 | 121 |
| >=5 <10 | 139 | 197 | 115 |
| >=10 | 153 | 256 | 120 |
| By BMI (kg/m^2) |  |  |  |
| <25 | 134 | 237 | 100 |
| >=25 <30 | 233 | 409 | 182 |
| >=30 <35 | 178 | 270 | 141 |
| >=35 | 70 | 303 | 24 |

CV death, cardiovascular death. CV death is defined as death from MI, Other IHD, heart failure or stroke (as in UKPDS-OM2). MAPE is the absolute percentage error averaged across the 7 years of follow-up.

## Appendix Table 7: Performance of UKPDS-OM2 at year 7 for participants in ASCEND validation cohort, for participants with complete baseline information (n = 7578)

|  | Observed cumulative incidence (%)  (95% CI) | Predicted^1^ | | | |  | Predicted^2^ | | | |
| --- | --- | --- | --- | --- | --- | --- | --- | --- | --- | --- |
| **Endpoints** |  | **Cumulative incidence (%)** | **% error** | **MAPE (%)** | **C-statistic** |  | **Cumulative incidence (%)** | **% error** | **MAPE (%)** | **C-statistic** |
| All-cause death | 9.6 (8.3, 10.8) | 20.0 | 109 | 213 | 0.73 |  | 18.2 | 90 | 200 | 0.73 |
| CV death | 1.8 (1.3, 2.4) | 8.0 | 335 | 382 | 0.73 |  | 6.1 | 231 | 318 | 0.74 |
| Other death | 7.7 (6.5, 8.9) | 12.0 | 55 | 167 | 0.72 |  | 12.1 | 56 | 168 | 0.73 |
| MI | 2.4 (1.8, 3.0) | 7.1 | 202 | 202 | 0.61 |  | 5.8 | 144 | 167 | 0.62 |
| Other IHD | 6.5 (5.4, 7.5) | 5.2 | -20 | 21 | 0.59 |  | 4.2 | -35 | 30 | 0.60 |
| Stroke | 2.8 (2.0, 3.5) | 4.3 | 53 | 68 | 0.67 |  | 3.4 | 22 | 49 | 0.66 |
| Heart failure | 1.5 (1.1, 2.1) | 3.9 | 151 | 196 | 0.68 |  | 3.1 | 98 | 167 | 0.69 |
| Amputation | 0.7 (0.3, 1.1) | 1.0 | 53 | 18 | 0.76 |  | 0.7 | -3 | 14 | 0.75 |

CV death, cardiovascular death; MI, myocardial infarction; Other IHD, other ischaemic heart disease; CI, confidence interval. CV death is defined as death from MI, Other IHD, heart failure or stroke (as in UKPDS-OM2). Percentage error is the degree of over-prediction as percentage of observed (negative values represent under-prediction) calculated using the cumulative incidences at year 7. MAPE is the absolute percentage error averaged across the 7 years of follow-up.

^1^UKPDS-OM2 risk factor progression equations used to project risk factor values during follow-up (base case).

^2^Values of risk factors during follow-up fixed to baseline values (sensitivity analysis).

## Appendix Table 8: Performance of UKPDS-OM2 at year 7 for participants in ASCEND validation cohort, for participants in placebo aspirin and placebo omega-3 fatty acid arm only (n = 3651)

|  | Observed cumulative incidence (%)  (95% CI) | Predicted^1^ | | | |  | Predicted^2^ | | | |
| --- | --- | --- | --- | --- | --- | --- | --- | --- | --- | --- |
| **Endpoints** |  | **Cumulative incidence (%)** | **% error** | **MAPE (%)** | **C-statistic** |  | **Cumulative incidence (%)** | **% error** | **MAPE (%)** | **C-statistic** |
| All-cause death | 9.6 (8.6, 10.6) | 18.8 | 97 | 133 | 0.69 |  | 17.0 | 78 | 122 | 0.69 |
| CV death | 2.2 (1.7, 2.7) | 7.5 | 248 | 255 | 0.64 |  | 5.6 | 162 | 205 | 0.65 |
| Other death | 7.4 (6.6, 8.3) | 11.3 | 53 | 96 | 0.71 |  | 11.4 | 54 | 97 | 0.71 |
| MI | 2.7 (2.2, 3.3) | 6.9 | 155 | 146 | 0.53 |  | 5.5 | 105 | 116 | 0.53 |
| Other IHD | 5.8 (5.1, 6.6) | 5.1 | -11 | 17 | 0.58 |  | 4.2 | -28 | 27 | 0.58 |
| Stroke | 2.9 (2.4, 3.5) | 4.0 | 37 | 29 | 0.70 |  | 3.1 | 8 | 12 | 0.70 |
| Heart failure | 1.1 (0.8, 1.6) | 3.6 | 218 | 265 | 0.78 |  | 2.8 | 149 | 228 | 0.77 |
| Amputation | 0.8 (0.6, 1.2) | 1.0 | 18 | 22 | 0.76 |  | 0.6 | -26 | 37 | 0.74 |

CV death, cardiovascular death; MI, myocardial infarction; Other IHD, other ischaemic heart disease; CI, confidence interval. CV death is defined as death from MI, Other IHD, heart failure or stroke (as in UKPDS-OM2). Percentage error is the degree of over-prediction as percentage of observed (negative values represent under-prediction) calculated using the cumulative incidences at year 7. MAPE is the absolute percentage error averaged across the 7 years of follow-up.

^1^UKPDS-OM2 risk factor progression equations used to project risk factor values during follow-up (base case).

^2^Values of risk factors during follow-up fixed to baseline values (sensitivity analysis).

## Appendix Table 9: Predicted lifetime cost, life expectancy and quality-adjusted life years from 15% risk reduction in cardiovascular death, based on risk predicted by the UKPDS-OM2 and observed in ASCEND

| Baseline 7-year risk of CV death | Lifetime cost (£) | | |  | QALYs | | |  | Life expectancy (years) | | |  | ICER (£ / QALY) |
| --- | --- | --- | --- | --- | --- | --- | --- | --- | --- | --- | --- | --- | --- |
|  | W/o tx | With tx | Incremental cost |  | W/o tx | With tx | Incremental QALYs |  | W/o tx | With tx | Incremental life years |  |  |
| **In entire cohort** |  |  |  |  |  |  |  |  |  |  |  |  |  |
| 2.0% (observed in ASCEND) | 28790 | 34927 | 6137 |  | 10.318 | 10.377 | 0.058 |  | 12.909 | 12.976 | 0.066 |  | 105540 |
| 7.5% (predicted by UKPDS-OM2) | 29233 | 34812 | 5579 |  | 9.252 | 9.470 | 0.218 |  | 11.692 | 11.941 | 0.249 |  | 25585 |
| **In lowest risk decile** |  |  |  |  |  |  |  |  |  |  |  |  |  |
| 1.1% (observed in ASCEND) | 32399 | 40075 | 7676 |  | 12.856 | 12.919 | 0.063 |  | 16.152 | 16.223 | 0.071 |  | 121573 |
| 1.7% (predicted by UKPDS-OM2) | 32497 | 40054 | 7558 |  | 12.626 | 12.724 | 0.098 |  | 15.894 | 16.004 | 0.109 |  | 77450 |
| **In highest risk decile** |  |  |  |  |  |  |  |  |  |  |  |  |  |
| 4.5% (observed in ASCEND) | 19530 | 23531 | 4001 |  | 6.727 | 6.792 | 0.065 |  | 8.405 | 8.482 | 0.077 |  | 61340 |
| 19.3% (predicted by UKPDS-OM2) | 20100 | 23332 | 3232 |  | 5.297 | 5.577 | 0.280 |  | 6.711 | 7.042 | 0.331 |  | 11552 |

W/o tx, with no treatment effect; With tx, with treatment effect i.e. 15% relative risk reduction applied; CV death, cardiovascular death; QALY, quality-adjusted life year; ICER, incremental cost-effectiveness ratio. CV death is defined as death from MI, Other IHD, heart failure or stroke (as in UKPDS-OM2). A hypothetical treatment that reduces CV death by 15% and costs UK £1.30/day was used. Costs, QALYs, life years, separately and as inputs into ICER, were discounted at 3.5% per annum. See Appendix Section 3 for further details about how these outcomes were estimated and the simulated scenarios.

# Appendix Figures

## Appendix Figure 1: Comparison of cumulative incidence of MI and Other IHD, predicted by the UKPDS-OM2 over 10 years with that observed in ASCEND, including and excluding coronary revascularizations


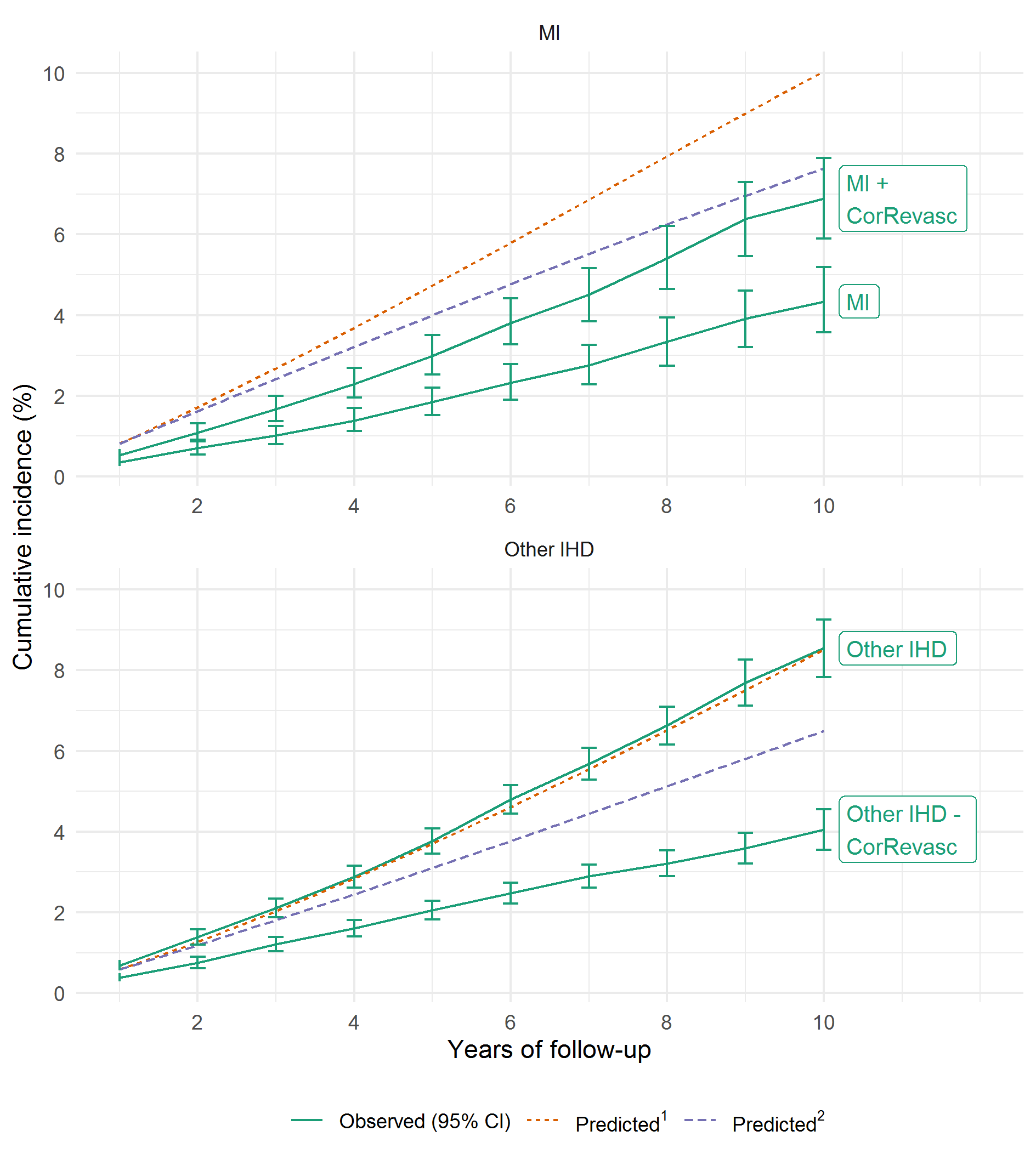


MI, myocardial infarction; MI + CorRevasc, myocardial infarction and coronary revascularization; Other IHD, other ischaemic heart disease (including coronary revascularization); Other IHD – CorRevasc, Other IHD excluding coronary revascularization; CI, confidence interval. The dotted lines represent the predicted cumulative incidence of MI and Other IHD (including coronary revascularization) in the respective plot.

^1^UKPDS-OM2 risk factor progression equations used to project risk factor values during (base case).

^2^Values of risk factor during follow-up fixed to baseline values (sensitivity analysis).

## Appendix Figure 2: Comparison of cumulative incidence of heart failure and amputation predicted by the UKPDS-OM2 over 10 years with that observed in ASCEND


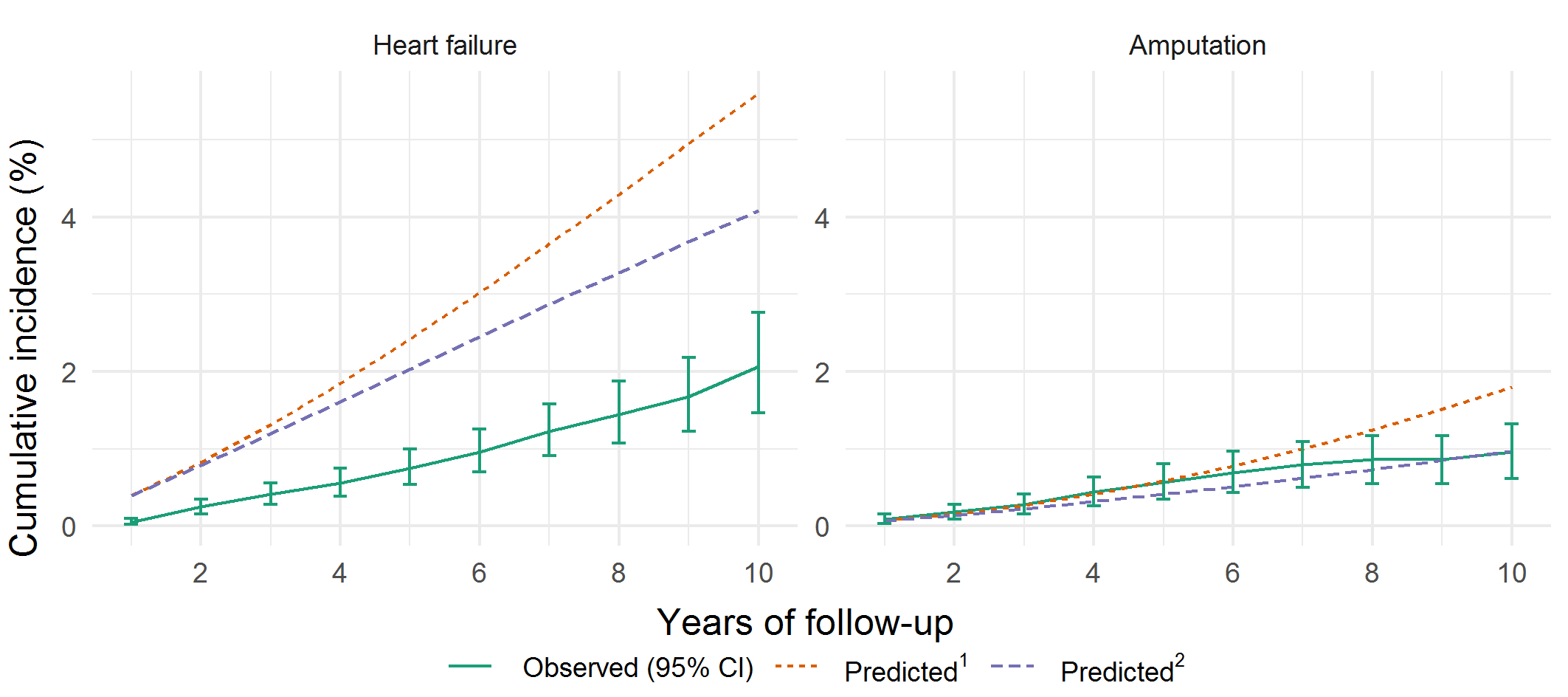


CI, confidence interval.

^1^UKPDS-OM2 risk factor progression equations used to project risk factor values during follow-up (base case).

^2^Values of risk factors during follow-up fixed to baseline values (sensitivity analysis).

## Appendix Figure 3: Comparison of 7-year cumulative incidence of complications predicted by the UKPDS-OM2 with that observed in ASCEND, stratified by decile of predicted 7-year cumulative incidence for each outcome


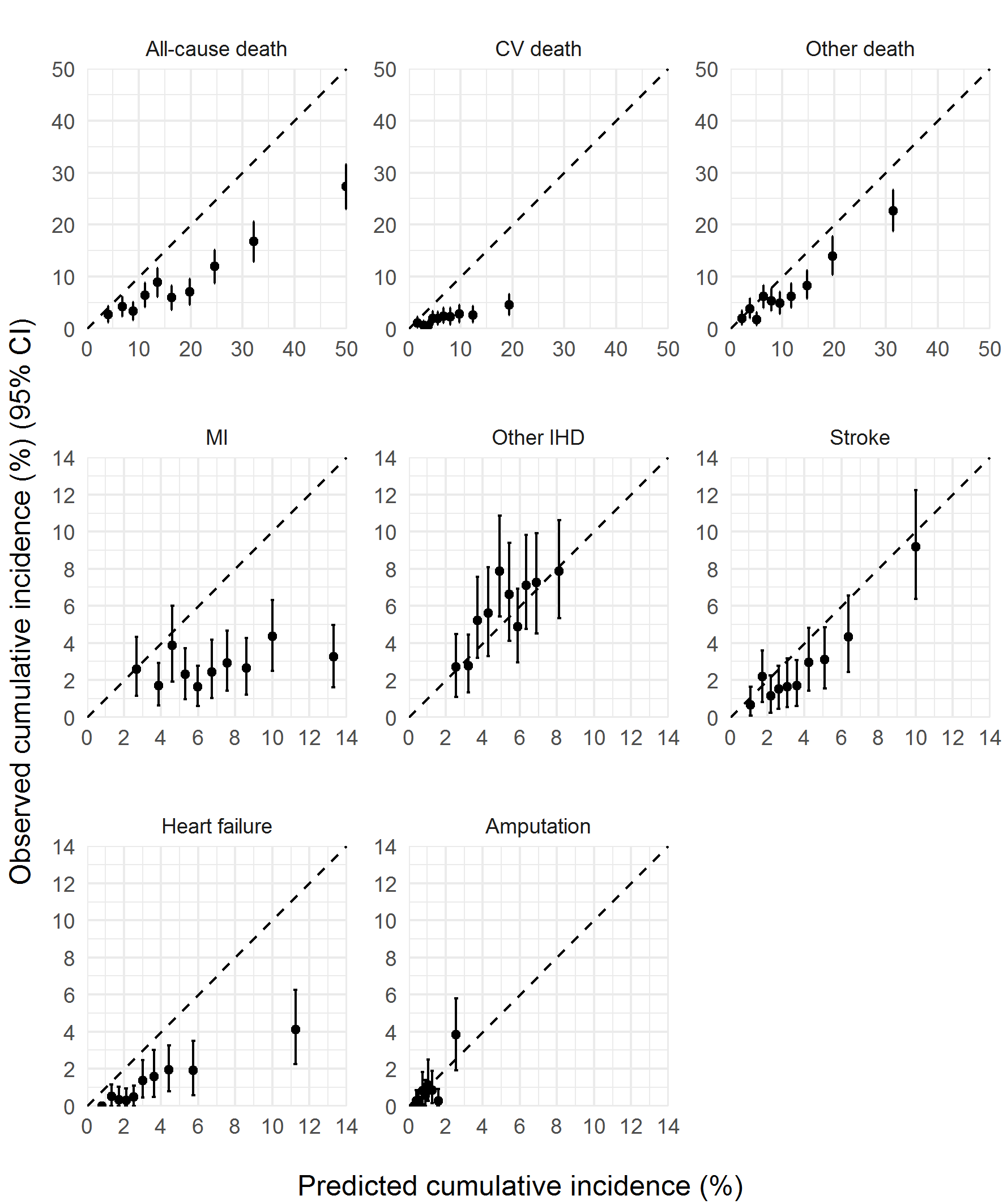


CV death, cardiovascular death; MI, myocardial infarction; Other IHD, other ischaemic heart disease; CI, confidence interval. CV death is defined as death from MI, Other IHD, heart failure or stroke (as in UKPDS-OM2).

45˚ line represents line of perfect correspondence between observed and predicted cumulative incidence.

## Appendix Figure 4: Comparison of cumulative incidence of complications predicted by the UKPDS-OM2 over 10 years with that observed in ASCEND, by sex


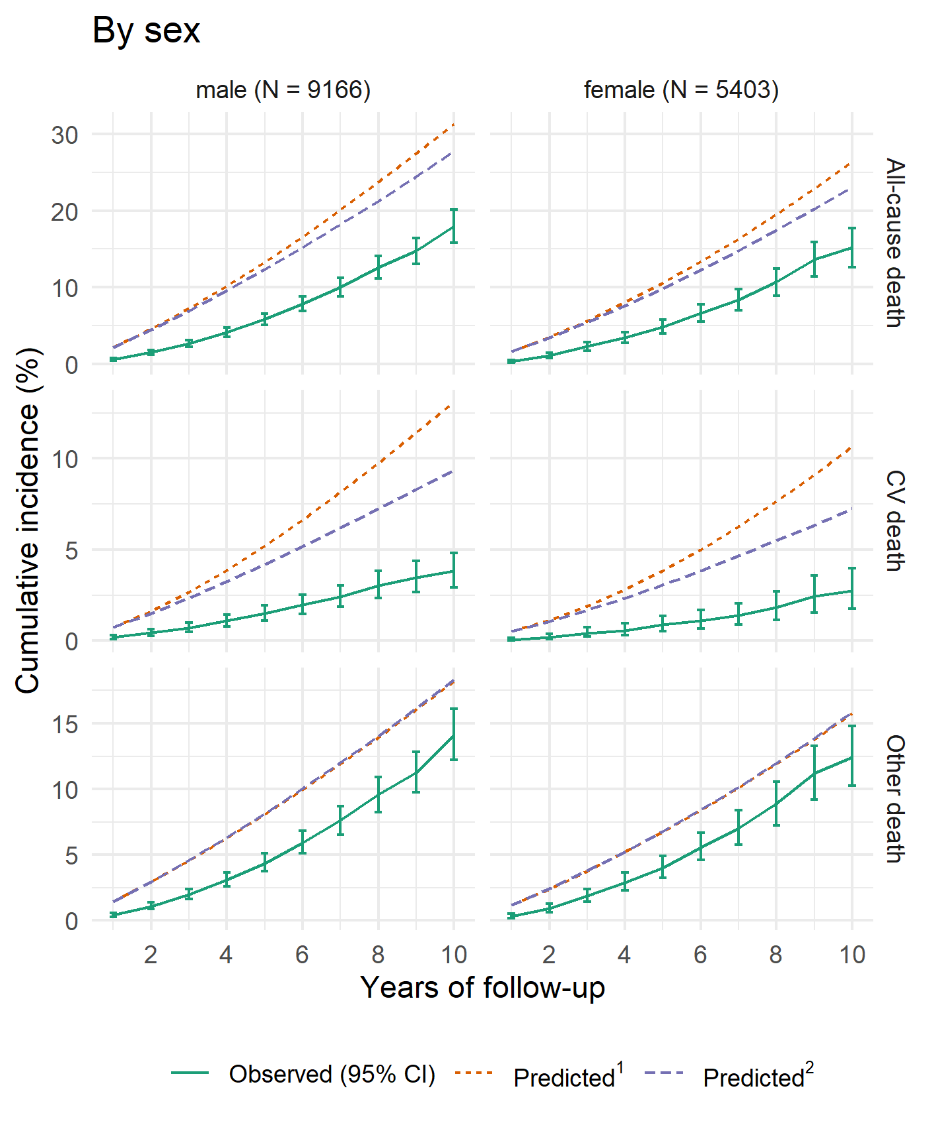


CV death, cardiovascular death. CV death is defined as death from MI, Other IHD, heart failure or stroke (as in UKPDS-OM2).

^1^UKPDS-OM2 risk factor progression equations used to project risk factor values during follow-up (base case).

^2^Values of risk factors during follow-up fixed to baseline values (sensitivity analysis).

## Appendix Figure 5: Comparison of cumulative incidence of complications predicted by the UKPDS-OM2 over 10 years with that observed in ASCEND, by age at baseline


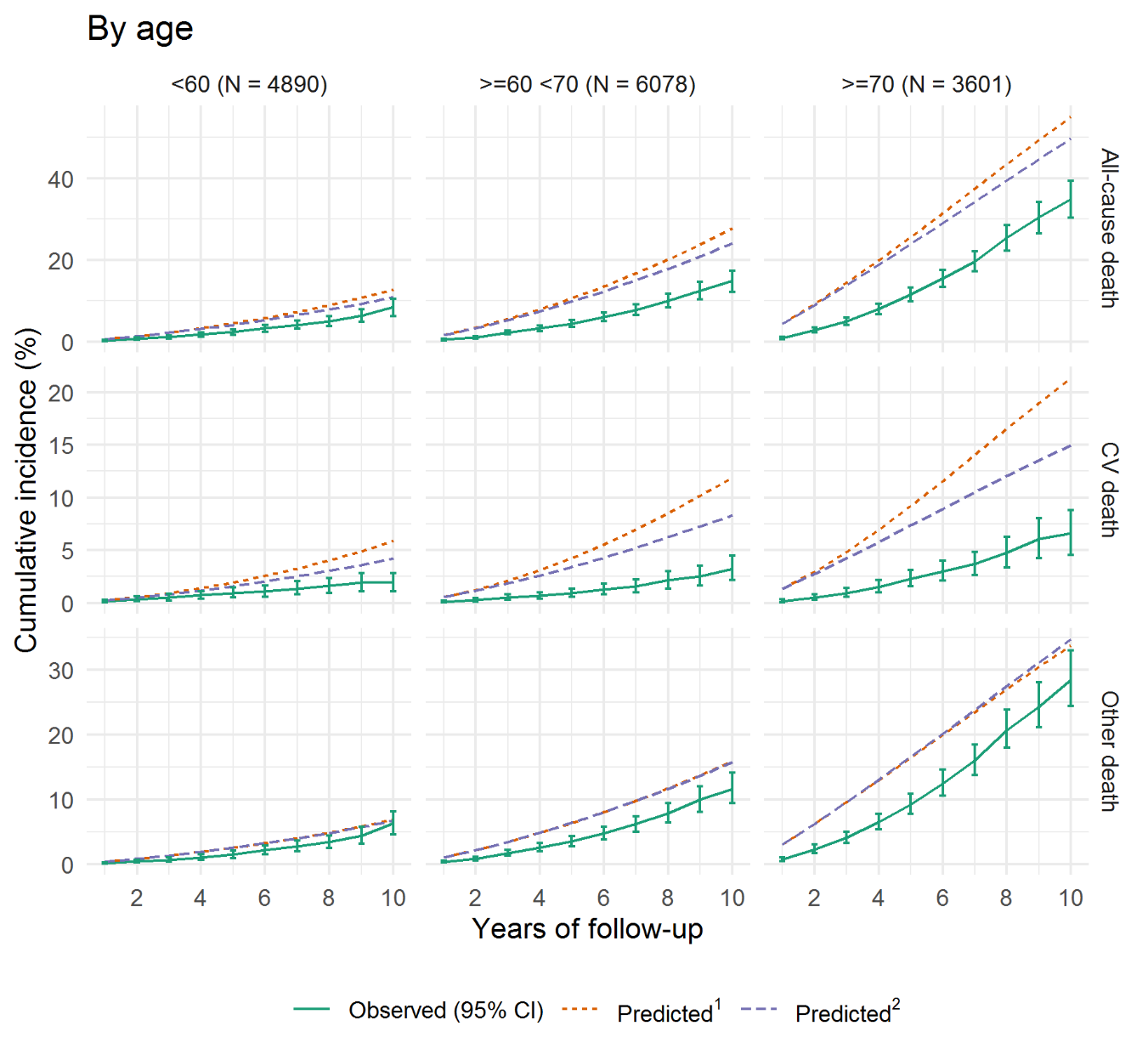


CV death, cardiovascular death. CV death is defined as death from MI, Other IHD, heart failure or stroke (as in UKPDS-OM2).

^1^UKPDS-OM2 risk factor progression equations used to project risk factor values during follow-up (base case).

^2^Values of risk factors during follow-up fixed to baseline values (sensitivity analysis).

## Appendix Figure 6: Comparison of cumulative incidence of complications predicted by the UKPDS-OM2 over 10 years with that observed in ASCEND, by HbA1c at baseline


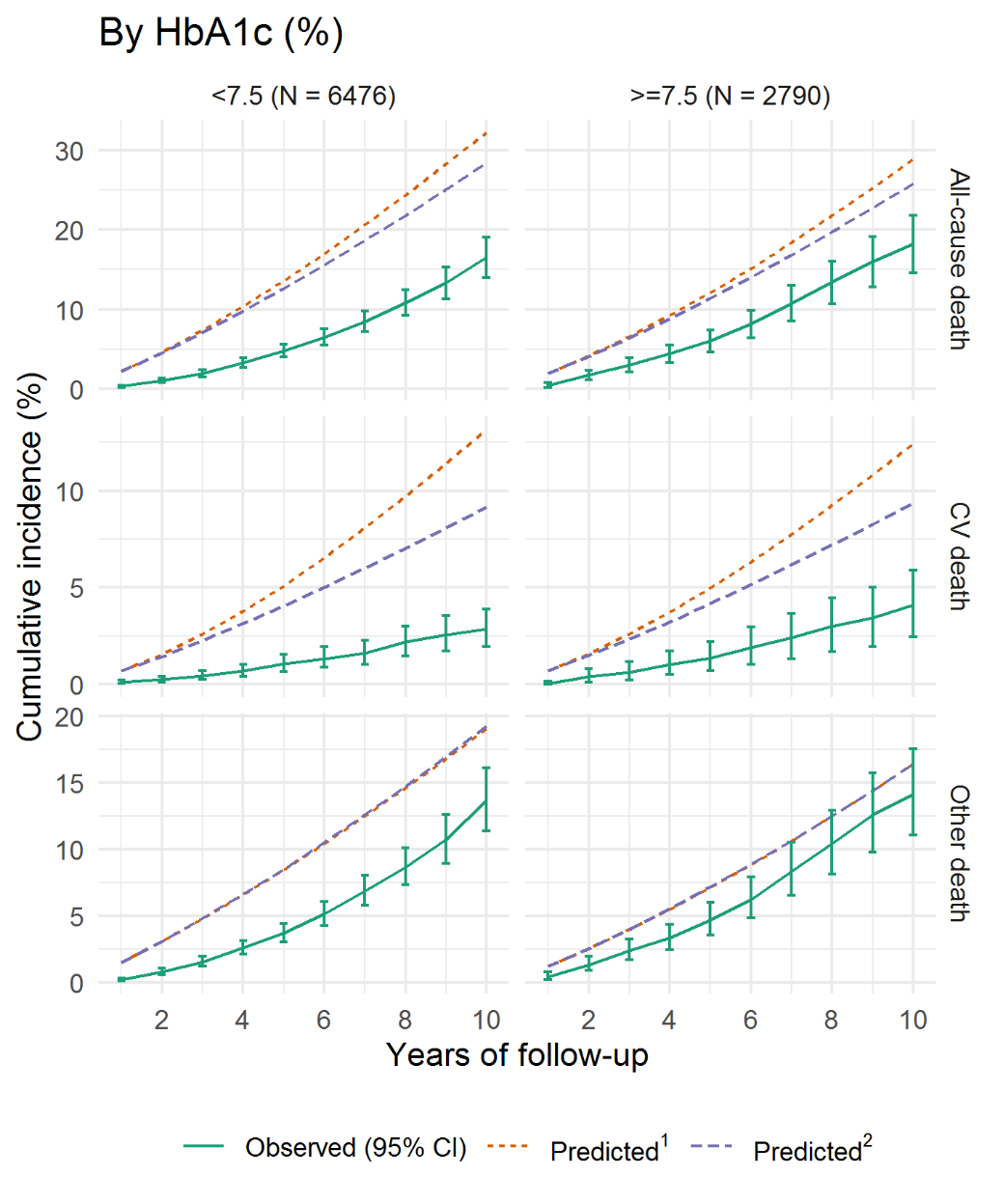


CV death, cardiovascular death. CV death is defined as death from MI, Other IHD, heart failure or stroke (as in UKPDS-OM2).

^1^UKPDS-OM2 risk factor progression equations used to project risk factor values during follow-up (base case).

^2^Values of risk factors during follow-up fixed to baseline values (sensitivity analysis).

## Appendix Figure 7: Comparison of cumulative incidence of complications predicted by the UKPDS-OM2 over 10 years with that observed in ASCEND, by duration of diabetes at baseline


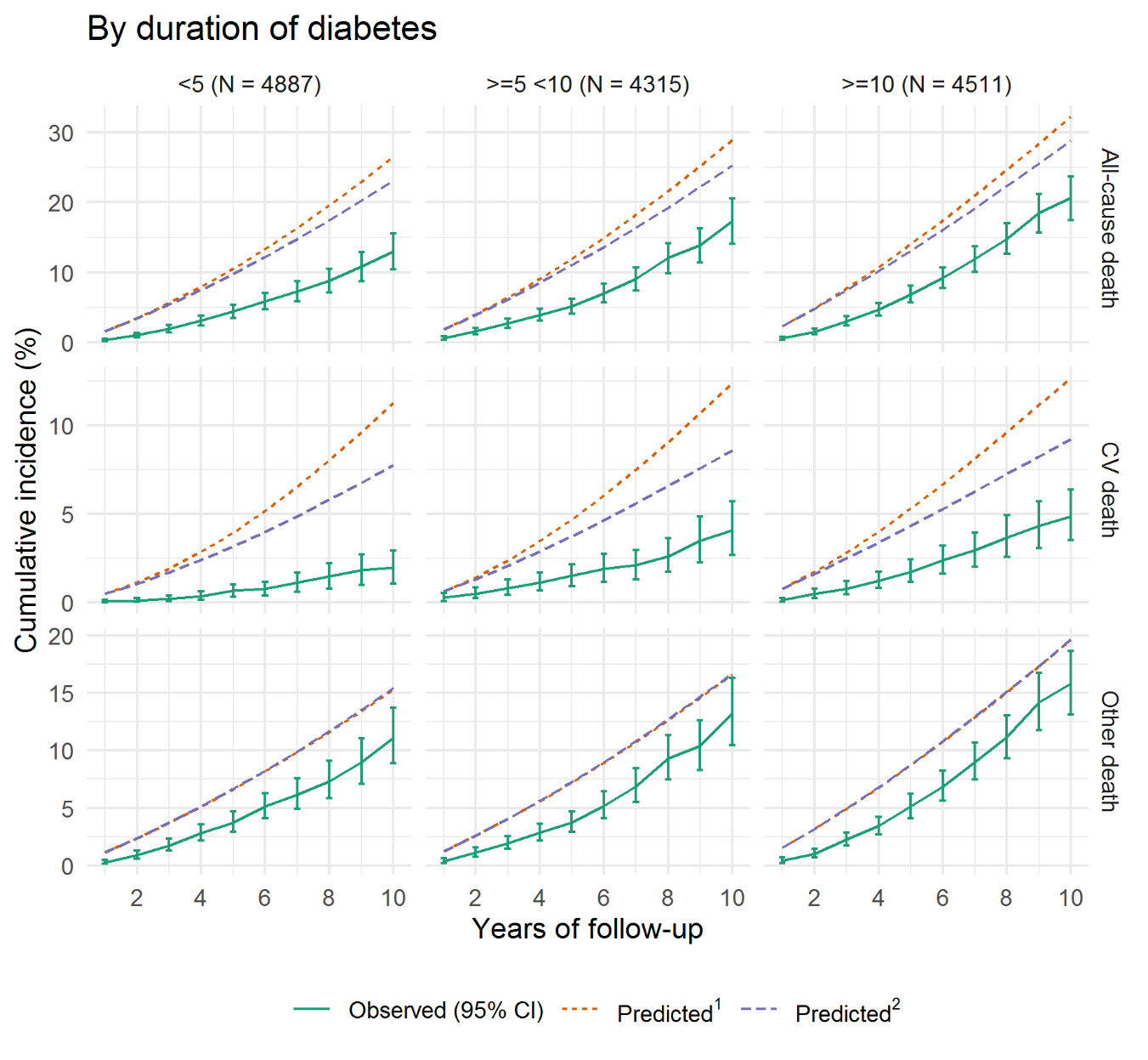


CV death, cardiovascular death. CV death is defined as death from MI, Other IHD, heart failure or stroke (as in UKPDS-OM2).

^1^UKPDS-OM2 risk factor progression equations used to project risk factor values during follow-up (base case).

^2^Values of risk factors during follow-up fixed to baseline values (sensitivity analysis).

## Appendix Figure 8: Comparison of cumulative incidence of complications predicted by the UKPDS-OM2 over 10 years with that observed in ASCEND, by BMI at baseline


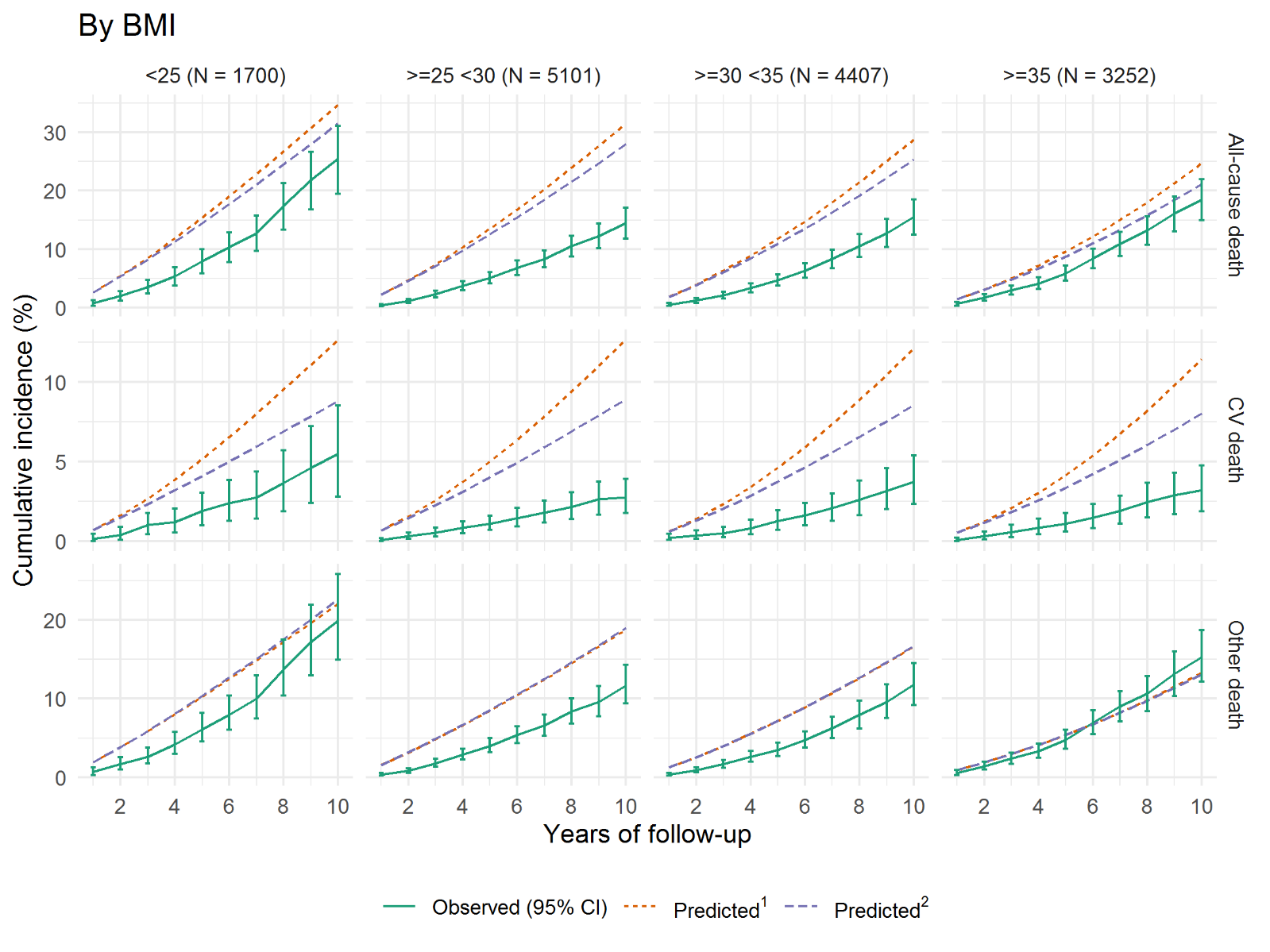


CV death, cardiovascular death. CV death is defined as death from MI, Other IHD, heart failure or stroke (as in UKPDS-OM2).

^1^UKPDS-OM2 risk factor progression equations used to project risk factor values during follow-up (base case).

^2^Values of risk factors during follow-up fixed to baseline values (sensitivity analysis).

## Appendix Figure 9: Comparison of cumulative incidence of complications predicted by the UKPDS-OM2 over 10 years with that observed in ASCEND, for participants with complete baseline information (N = 7578)


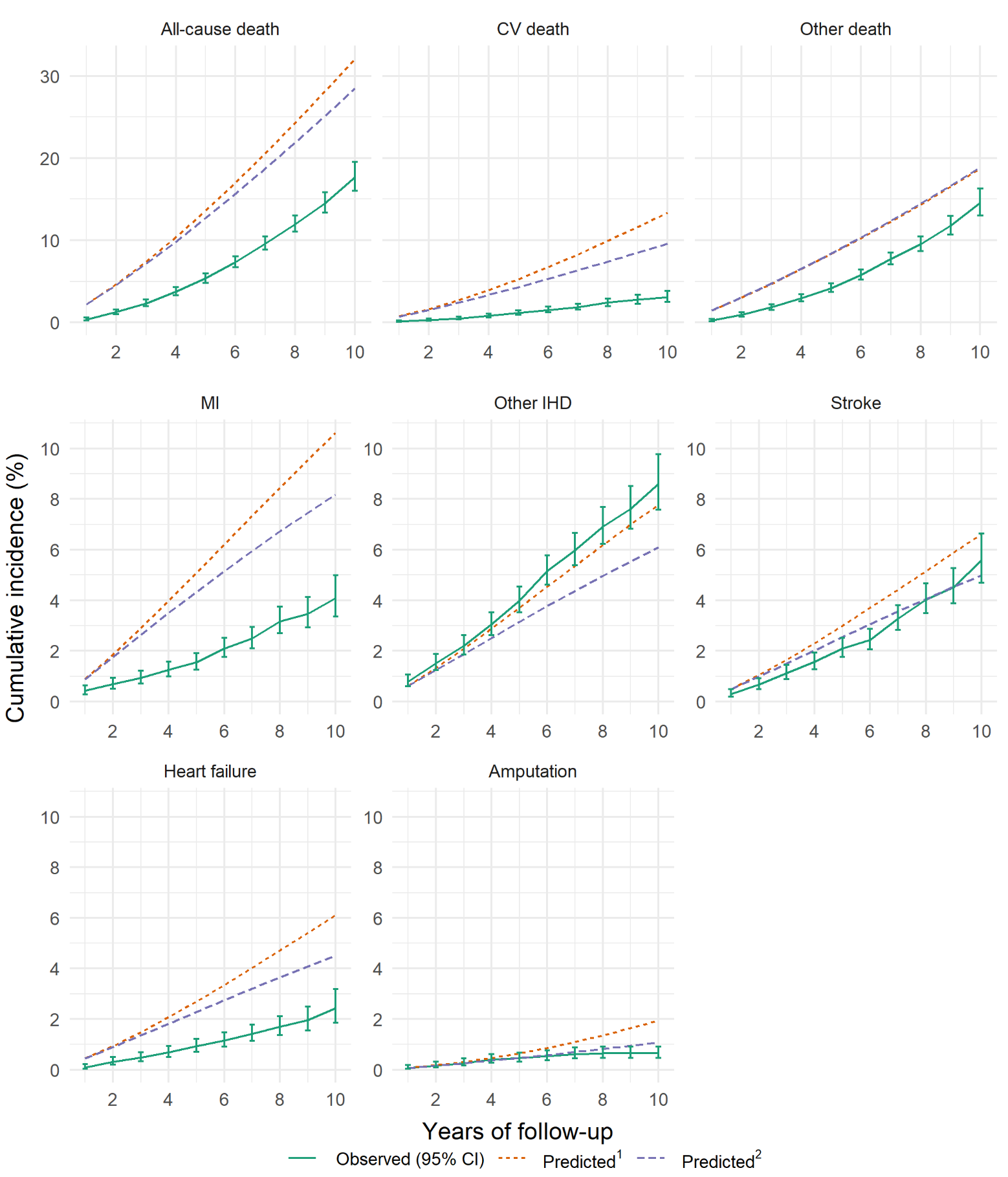


CV death, cardiovascular death; MI, myocardial infarction; Other IHD, other ischaemic heart disease; CI, confidence interval. CV death is defined as death from MI, Other IHD, heart failure or stroke (as in UKPDS-OM2).

^1^UKPDS-OM2 risk factor progression equations used to project risk factor values during follow-up (base case).

^2^Values of risk factors during follow-up fixed to baseline values (sensitivity analysis).

## Appendix Figure 10: Comparison of cumulative incidence of complications predicted by the UKPDS-OM2 over 10 years with that observed in ASCEND, for participants in aspirin placebo and omega-3 placebo arm only (N = 3651)


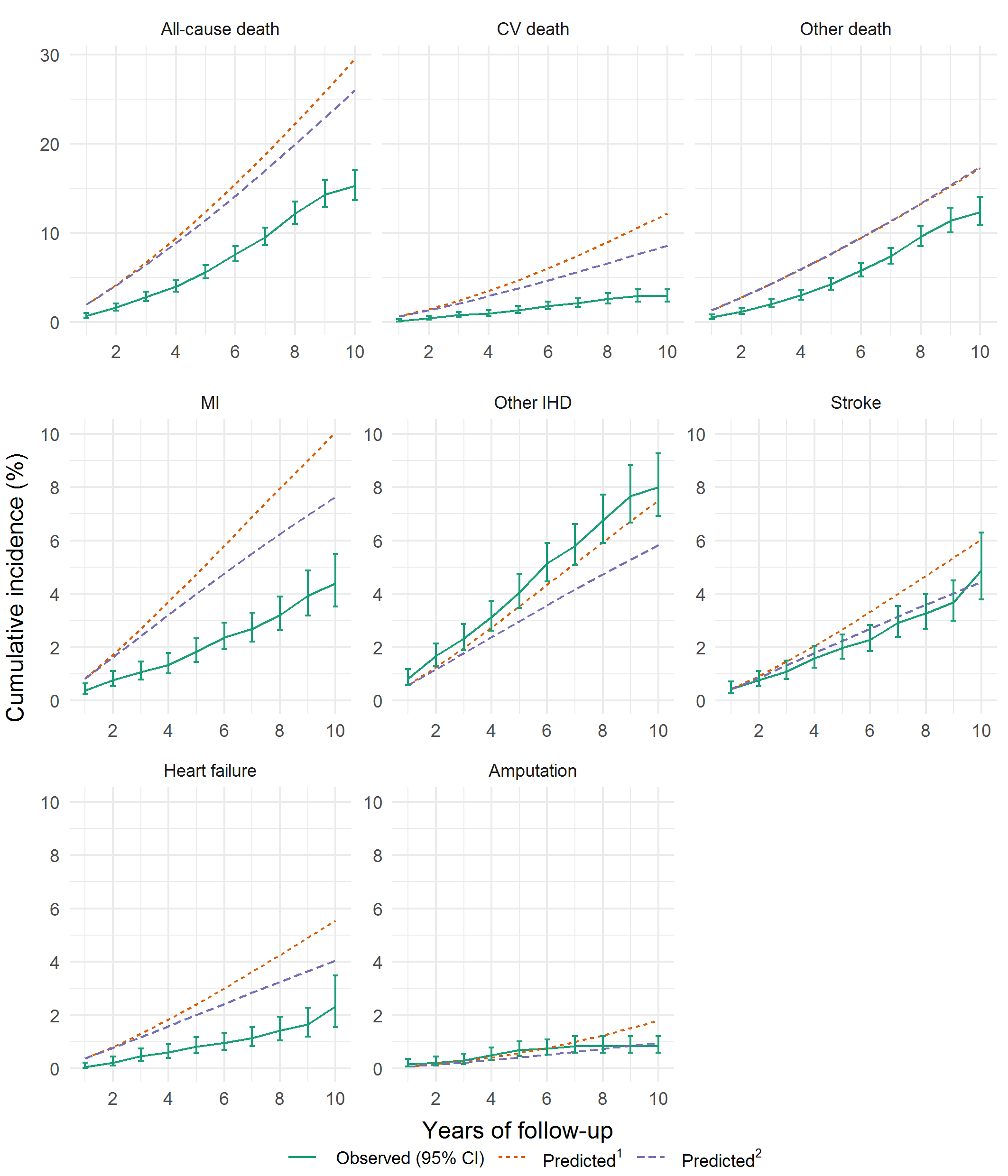


CV death, cardiovascular death; MI, myocardial infarction; Other IHD, other ischaemic heart disease; CI, confidence interval. CV death is defined as death from MI, Other IHD, heart failure or stroke (as in UKPDS-OM2).

^1^UKPDS-OM2 risk factor progression equations used to project risk factor values during follow-up (base case).

^2^Values of risk factors during follow-up fixed to baseline values (sensitivity analysis).

## Appendix Figure 11: Average values of risk factors predicted by the UKPDS risk factor equations (solid black line) compared to those observed (marked ‘X’) in a random sample of ASCEND participants^†^


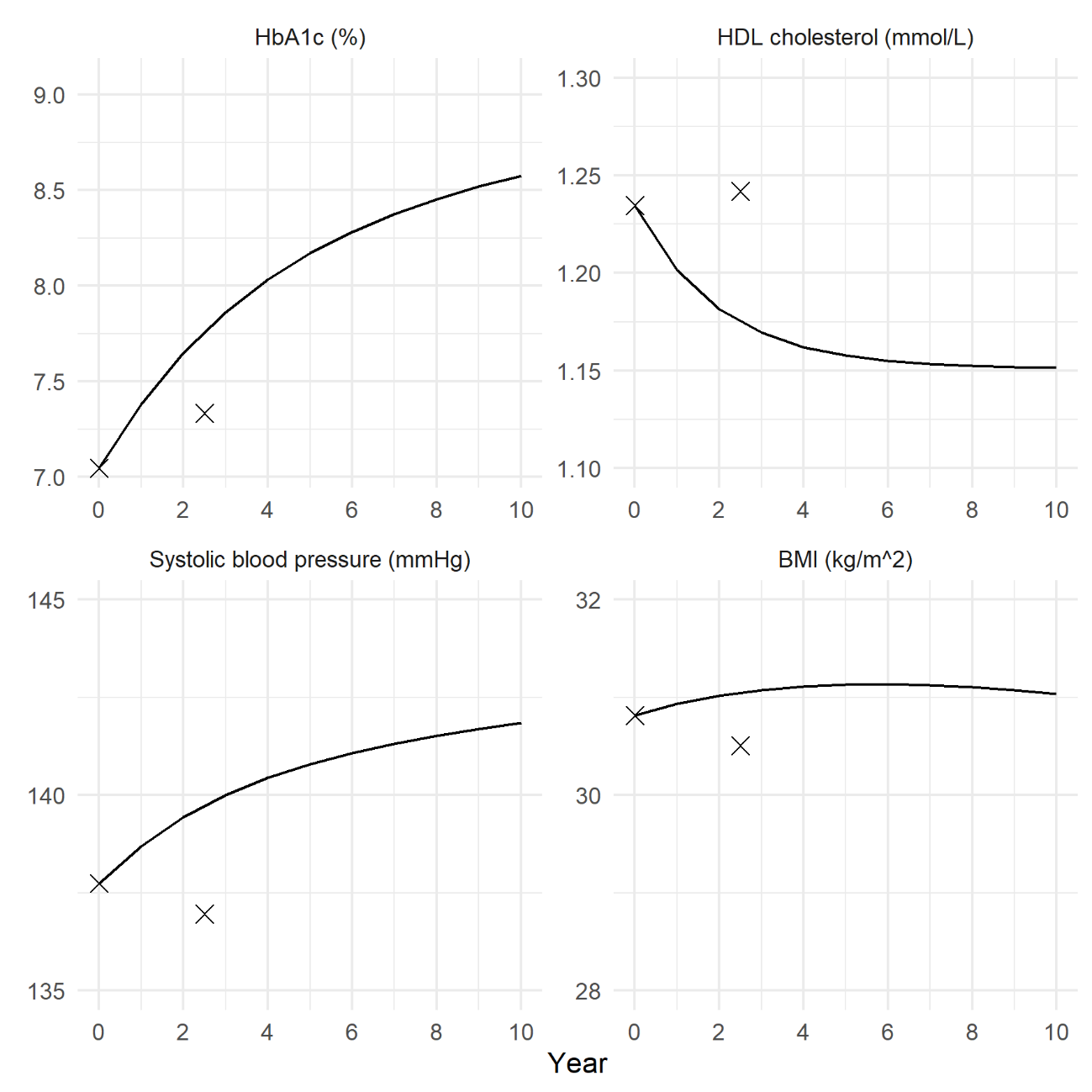


HbA1c, glycated haemoglobin; HDL, high density lipoprotein. The solid black line is the average value predicted by the risk factor progression equations in UKPDS Outcomes Model 2, while the ‘X’ marks are the average values observed in ASCEND.

^†^Average values of risk factor observed in the random sample of ASCEND participants who returned blood/urine sample at average 2.5 years into follow-up [N = 1249 (9%) in the validation cohort].

# Appendix Section 1: Missing baseline data

**Imputing values for biomakers not available in ASCEND**

Patient-level data from the UKPDS^1^ was used to develop linear regression models to predict haemoglobin and white blood cell counts (WBC) using other patient characteristics and biomarkers. Variables were selected using stepwise selection with p-values from Chi-squared test as the criteria for adding (p < 0.05) or dropping (p < 0.01) variables. Both WBC and haemoglobin were log-transformed as the quantile-quantile plots for both linear models demonstrated skewed residuals, and this was confirmed with a Box-Cox test with the optimal $\lambda$ close to 0. For predicting haemoglobin levels, variables in final model include gender, ethnicity, smoking status, duration of diabetes, LDL cholesterol, systolic blood pressure [SBP], diastolic blood pressure [DBP], HbA1c and eGFR. For predicting WBC levels, variables in final model include ethnicity, smoking status, BMI, HDL cholesterol, LDL cholesterol, SBP, DBP, HbA1c and presence of microalbuminuria.

Statins lower LDL cholesterol, and about 75% of ASCEND participants were using statins. Hence, the UKPDS data was deemed unsuitable for estimating LDL cholesterol from other biomarkers as statin use was much less common at recruitment into UKPDS. Instead, patient-level data from the REVEAL study^2^ was used to develop linear regression model to predict LDL cholesterol using other biomarkers, particularly Apo(B) which is highly correlated with LDL cholesterol (variables in final model include HDL cholesterol, non-HDL cholesterol, Apo(B), gender, age, smoking status, SBP, DBP, eGFR, presence of diabetes). In REVEAL, all patients were on statin treatment. The model was fitted using all patients, with an indicator for diabetes patients to reflect the effect of diabetes on LDL cholesterol. Similar coefficients were obtained in a model fitted in the subset of participants with diabetes in REVEAL (37%).

**Multiple imputation for missing data in ASCEND**

Excluding data not available in ASCEND, 51% of participants had at least one missing variable. 60 imputed datasets were generated using a multivariate imputation by chained equations (MICE) method, with predictive mean matching method used for continuous variables and logistic regression imputation used for binary variables.^3^

Two approaches were undertaken for simulation:

1. Event occurrence were predicted for all participants in each of the 60 imputed datasets and then results were pooled across;
2. For each missing value of each patient, the mean (for continuous variables) / most frequent (for binary variables) value across the 60 imputed datasets is used to establish a single dataset with imputed data. Event occurrence were predicted for participants in this single dataset.

Results from the two approaches were very similar (see figure below). Hence, the results from approach 2 are presented throughout the manuscript.


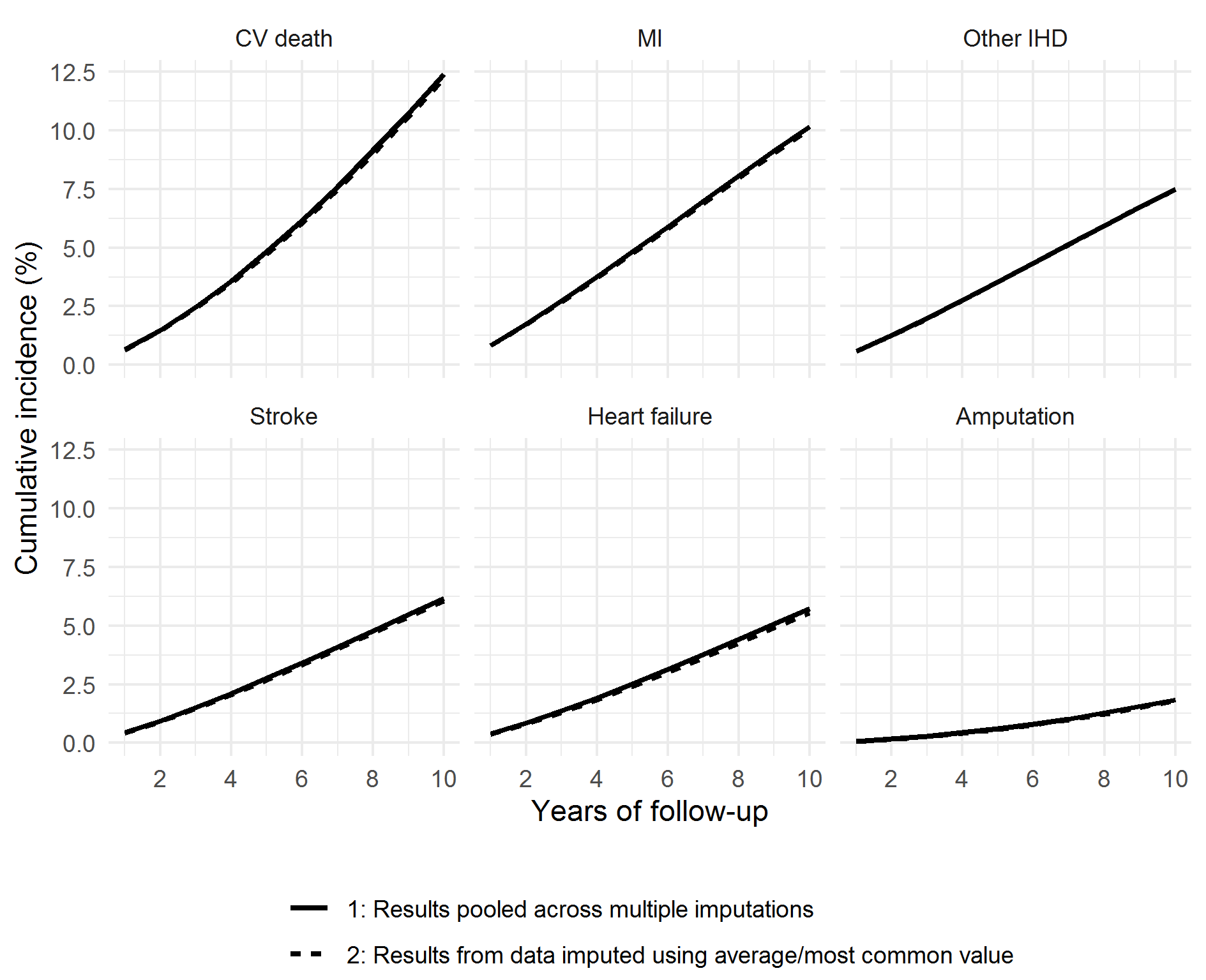


CV death, cardiovascular death; MI, myocardial infarction; Other IHD, other ischaemic heart disease. CV death is defined as death from MI, Other IHD, heart failure or stroke (as in UKPDS-OM2). Results above are for participants in ASCEND allocated to the aspirin placebo and omega-3 fatty acid placebo arm only.

**References**

1. Holman RR, Paul SK, Bethel MA, et al. 10-Year Follow-up of Intensive Glucose Control in Type 2 Diabetes. *N Engl J Med* 2008; 359: 1577–1589.

2. The HPS3/TIMI55–REVEAL Collaborative Group. Effects of Anacetrapib in Patients with Atherosclerotic Vascular Disease. *N Engl J Med* 2017; 377: 1217–1227.

3. White IR, Royston P, Wood AM. Multiple imputation using chained equations: Issues and guidance for practice. *Statistics in Medicine* 2011; 30: 377–399.

# Appendix Section 2: TRIPOD statement

| **Section/Topic** | **Item** | **Checklist Item** | **Page** |
| --- | --- | --- | --- |
| **Title and abstract** | | | |
| Title | 1 | Identify the study as developing and/or validating a multivariable prediction model, the target population, and the outcome to be predicted. | 1 |
| Abstract | 2 | Provide a summary of objectives, study design, setting, participants, sample size, predictors, outcome, statistical analysis, results, and conclusions. | 2 |
| **Introduction** | | | |
| Background and objectives | 3a | Explain the medical context (including whether diagnostic or prognostic) and rationale for developing or validating the multivariable prediction model, including references to existing models. | 3-4 |
|  | 3b | Specify the objectives, including whether the study describes the development or validation of the model or both. | 4 |
| **Methods** | | | |
| Source of data | 4a | Describe the study design or source of data (e.g., randomized trial, cohort, or registry data), separately for the development and validation data sets, if applicable. | 4 |
|  | 4b | Specify the key study dates, including start of accrual; end of accrual; and, if applicable, end of follow-up. | 4 |
| Participants | 5a | Specify key elements of the study setting (e.g., primary care, secondary care, general population) including number and location of centres. | 4 – full details of ASCEND study published elsewhere and referenced |
|  | 5b | Describe eligibility criteria for participants. | 4 |
|  | 5c | Give details of treatments received, if relevant. | 4 |
| Outcome | 6a | Clearly define the outcome that is predicted by the prediction model, including how and when assessed. | 5 |
|  | 6b | Report any actions to blind assessment of the outcome to be predicted. | Assessment is unblinded |
| Predictors | 7a | Clearly define all predictors used in developing or validating the multivariable prediction model, including how and when they were measured. | 4; Appendix Table 1 |
|  | 7b | Report any actions to blind assessment of predictors for the outcome and other predictors. | NA |
| Sample size | 8 | Explain how the study size was arrived at. | 4 |
| Missing data | 9 | Describe how missing data were handled (e.g., complete-case analysis, single imputation, multiple imputation) with details of any imputation method. | 5; Appendix Section 1 |
| Statistical analysis methods | 10c | For validation, describe how the predictions were calculated. | 6 |
|  | 10d | Specify all measures used to assess model performance and, if relevant, to compare multiple models. | 6 |
|  | 10e | Describe any model updating (e.g., recalibration) arising from the validation, if done. | NA - Model not re-calibrated |
| Risk groups | 11 | Provide details on how risk groups were created, if done. | 6 |
| Development vs. validation | 12 | For validation, identify any differences from the development data in setting, eligibility criteria, outcome, and predictors. | 9-10; Appendix Table 2; Appendix Table 4 |
| **Results** | | | |
| Participants | 13a | Describe the flow of participants through the study, including the number of participants with and without the outcome and, if applicable, a summary of the follow-up time. A diagram may be helpful. | 4 – full details of ASCEND study published elsewhere and referenced |
|  | 13b | Describe the characteristics of the participants (basic demographics, clinical features, available predictors), including the number of participants with missing data for predictors and outcome. | 7; Table 1 |
|  | 13c | For validation, show a comparison with the development data of the distribution of important variables (demographics, predictors and outcome). | Appendix Table 4 |
| Model performance | 16 | Report performance measures (with CIs) for the prediction model. | 7-8; Table 2; Appendix Table 5 – 7; Figure 1; Appendix Figure 1 – 10 |
| Model-updating | 17 | If done, report the results from any model updating (i.e., model specification, model performance). | NA - Model not re-calibrated |
| **Discussion** | | | |
| Limitations | 18 | Discuss any limitations of the study (such as nonrepresentative sample, few events per predictor, missing data). | 10-11 |
| Interpretation | 19a | For validation, discuss the results with reference to performance in the development data, and any other validation data. | 9-10 |
|  | 19b | Give an overall interpretation of the results, considering objectives, limitations, results from similar studies, and other relevant evidence. | 12 |
| Implications | 20 | Discuss the potential clinical use of the model and implications for future research. | 12 |
| **Other information** | | | |
| Supplementary information | 21 | Provide information about the availability of supplementary resources, such as study protocol, Web calculator, and data sets. | NA |
| Funding | 22 | Give the source of funding and the role of the funders for the present study. | Title page |

We recommend using the TRIPOD Checklist in conjunction with the TRIPOD Explanation and Elaboration document.

# Appendix Section 3: Estimating impact of over-predicting CV death risk on cost-effectiveness

Lifetime healthcare costs, life expectancies and quality-adjusted life years (QALYs) were estimated for each ASCEND participant using the UKPDS-OM2, with the default costs and quality of life utility values provided in the UKPDS-OM2 used.^1^ All outcomes were discounted at 3.5% per annum. The relationship between 7-year risk of CV death and lifetime healthcare costs, life expectancies, and QALYs predicted by the UKPDS-OM2 were estimated using linear regression models. Models included covariates sex, age, 7-year CV death risk and the interaction between age and 7-year CV death risk. Natural cubic splines were used to model the non-linear relationship between costs/life expectancy/QALYs and age. The fitted models were used to predict these outcomes in the ASCEND cohort, whereby the 7-year CV death risks for all participants in the ASCEND cohort were adjusted to simulate impact of over-predicting CV death risk on cost-effectiveness:

1. UKPDS-OM2 predicted risks scenario
   1. CV death risk for each participant as predicted by the UKPDS-OM2 (i.e. over-estimated risk)
   2. 15% relative risk reduction in risk of CV death applied to risk to (a)
2. ASCEND risks scenario
   1. CV death risk for each participant predicted by the UKPDS-OM2 scaled by a scaling factor (see Appendix Table 10) so mean risk across cohort matches the risk observed in ASCEND
   2. 15% relative reduction in risk of CV death applied to risk in (a)

Results from (1) were used to estimate the incremental cost-effectiveness ratio (ICER) for the scenario where CV death risk is over-estimated by the UKPDS-OM2. Results from (2) were used to estimate the ICER for the scenario where CV death risk corresponds to risk observed in ASCEND. The estimated lifetime costs in scenarios with added treatment further included an illustrative cost of treatment of £1.30 per day. These calculations were also performed separately among ASCEND participants in the lowest CV death risk decile and in the highest CV death risk decile respectively.

*Appendix* *Table 10: Scaling factor applied to 7-year CV death risk predicted by UKPDS-OM2 to match risk observed in ASCEND*

|  | Risk observed in ASCEND | Risk predicted by UKPDS-OM2 | Scaling factor |
| --- | --- | --- | --- |
| All participants in ASCEND cohort | 2.0% | 7.5% | 0.267 |
| Lowest CV death risk decile | 1.1% | 1.7% | 0.647 |
| Highest CV death risk decile | 4.5% | 19.3% | 0.233 |

**References**

1. UKPDS Outcomes Model User Manual. University of Oxford Diabetes Trials Unit and Health Economics Research Centre; 2015. Available from: https://www.dtu.ox.ac.uk/outcomesmodel/OM2Manual.pdf
